# A 35-Year Review of Pre-Clinical HIV Therapeutics Research Reported by NIH ChemDB: Influences of Target Discoveries, Drug Approvals and Research Funding

**Published:** 2020-11-18

**Authors:** Shawn S. Jackson, Louise E. Sumner, Mikaela A. Finnegan, Emily A. Billings, Danna L. Huffman, Margaret A. Rush

**Affiliations:** 1 Gryphon Scientific, LLC, Takoma Park, MD, USA; 2 GDIT, Falls Church, VA, USA

**Keywords:** HIV, AIDS, Therapeutics, Historical trends, Research funding, Drug development

## Abstract

We present a retrospective analysis of trends in human immunodeficiency virus (HIV) small molecule drug development over the last thirty-five years based on data captured by ChemDB, a United States (US) National Institutes of Health (NIH) database of chemical and biological HIV testing data. These data are analyzed alongside NIH funding levels, US Food and Drug Administration (FDA) drug approvals, and new target identifications to explore the influences of these factors on anti-HIV drug discovery research. The NIH’s ChemDB database collects chemical and biological testing data describing published and patented pre-clinical compounds in development as potential HIV therapeutics. These data were used as a proxy for estimating overall levels of HIV therapeutics research activities in order to assess research trends. Data extracted from ChemDB were compared with records of drug approvals from the FDA, NIH funding levels, and drug target discoveries to elucidate the influences that these factors have on levels of HIV therapeutics research activities. Despite the increasingly wide suite of HIV therapeutic options that have accumulated during decades of research, interest in HIV therapeutics research activities remains strong. While decreases in research activity levels have followed cuts in research funding, FDA-approved HIV therapeutics have continued to accumulate. The comparisons presented here indicate that HIV drug research activity levels have historically been more responsive to changes in funding levels and the identification of new drug targets, than they have been to drug approvals. Continued interest in HIV therapeutics research may reflect that fact that of the 55 drugs approved for HIV treatment as of 2018, only seven inhibitory targets are represented. Moreover, drug resistance presents substantial clinical challenges. Sustained research interest despite drug approvals and fluctuations in available funding likely reflects the clinical need for safer, more palatable and more efficacious therapeutics; robust attention to both novel therapeutics and inhibitory targets is necessary given the speed of development of drug-resistant HIV strains. Only with such continued interest will we reduce the burden of acquired immunodeficiency syndrome (AIDS) disease and control the AIDS epidemic.

## Introduction

**Figure 1.**
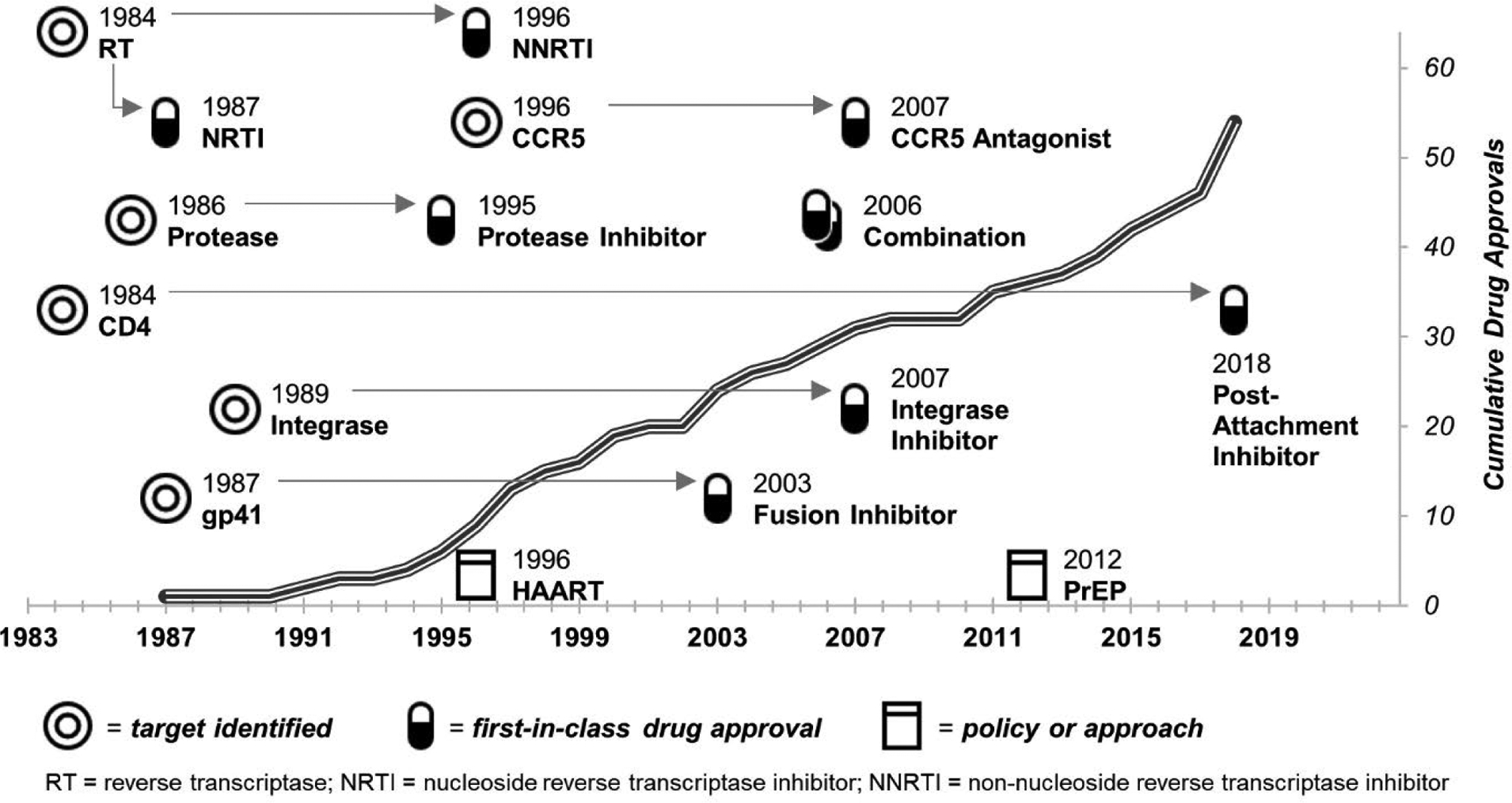
Timeline of HIV therapeutics target discoveries, first-in-class drug approvals, and major advances in treatment approaches. **Note:** Only those therapeutics for which an FDA approved drug(s) exists are included. Major shifts in clinical approaches to treatment and prevention are noted as HAART (highly active antiretroviral therapy) and PrEP (pre-exposure prophylaxis).

Thirty-five years have passed since the identification of human immunodeficiency virus (HIV) as the etiological agent of acquired immunodeficiency syndrome (AIDS) [1,2]. Over that time, billions of dollars have been spent on the development of HIV therapeutics in the hopes of reducing HIV-related morbidity and mortality, and someday achieving a cure. While the first therapeutic for the treatment of HIV-infected individuals was approved by the US Food and Drug Administration (FDA) in 1987 [3], the highly mutable biology of HIV quickly led to the emergence of drug-resistant strains [4,5], and additional drugs were soon needed to control the epidemic. Since 1987, dozens of HIV therapeutics have been developed (Figure 1) [3].

With the advent of effective antiretroviral treatment regimens including highly active antiretroviral therapy (HAART) [6], lifespans have increased dramatically for patients infected with HIV, and HIV infection has come to be viewed as a chronic disease state [7–9]. Therefore, maximizing the safety and tolerability of HIV treatment regimens has become of utmost importance. However, resistant viral strains appear quickly following the introduction of each new antiretroviral drug class, and so the need for antiretroviral therapies that reduce the likelihood of drug resistance is of equal, if not greater, importance. A recent World Health Organization (WHO) survey found that resistance is on the rise, with over 10% of people starting antiretroviral therapy in 6 of 11 African, Asian, and Latin American countries already having a drug-resistant strain of HIV. With pre-treatment drug resistance levels currently topping 10%, global targets to end AIDS as a public health threat by 2030 are unlikely to be met without rapid advances in antiretroviral therapy [10].

As many challenges face antiretroviral therapeutics development, an examination of historical influences and indicators that helps us better understand past developmental trends and milestones is warranted. We undertook a focused study of influences on the direction and pace of the HIV drug development field, analyzing the relationships between the quantities of therapeutic targets identified and potential HIV therapeutics tested in pre-clinical studies, levels of funding for such research, and the achieving of HIV drug approvals by the FDA. For this analysis, we leveraged the ChemDB HIV, Opportunistic Infection and Tuberculosis Therapeutics Database (ChemDB; chemdb.niaid.nih.gov) maintained by the National Institute of Allergy and Infectious Diseases (NIAID) of the National Institutes of Health (NIH). ChemDB contains information extracted from the scientific literature and patents on the structure and activity of pre-clinical compounds with potential therapeutic activity against HIV. In addition to their utility for a variety of drug discovery purposes, from virtual screening and structure-activity relationship (SAR) studies to building drug discovery models based on machine learning [11], ChemDB data are also useful for tracking HIV drug discovery activities more broadly. By querying the database, annual counts of compounds tested for anti-HIV activity were gathered, along with information on their novelty, source publications and potential viral and host targets of inhibition. Taken together, these data can serve as proxies for the relative levels of HIV therapeutic “research activities” taking place each year. As such, they can be used to draw inferences regarding the influences of factors such as funding levels and drug approvals on the extent and focus of therapeutics research activities. With this knowledge, we may better predict the combinations of factors that will support robust HIV drug discovery and development research in the future; this knowledge could be leveraged in future policy decisions, possibly leading to a faster attainment of antiretroviral therapeutics goals.

## Materials and Methods

### ChemDB

Data for 1984-June 2018 were obtained from the ChemDB HIV, Opportunistic Infection and Tuberculosis Therapeutics Database. (Data for July-December 2018 were not available at the time of this writing.) Since the database is continually updated with information extracted from published literature and patents, including the structure and activity of compounds that have been tested against HIV, HIV enzymes or opportunistic pathogens, it was considered to be a comprehensive data source. See the Appendix for literature surveillance methodology. The following data were extracted for each year: (1) the total number of putative HIV-inhibitory compounds described, including both those previously existing in and novel to ChemDB; (2) the number of unique viral or host cellular components investigated as targets for HIV inhibition (e.g., reverse transcriptase (RT), protease, integrase, CCR5, CD4, etc.); (3) the number of publications (or patents) describing a compound(s) with putative activity against HIV; and (4) the total number of references to targets (“target references”) for HIV inhibition within the publications (or patents). Note that a single publication/patent could describe multiple potential inhibitory compounds and reference multiple targets. Testing data that did not specify an inhibitory target and studies for which latent HIV reactivation was the goal were excluded from analyses.

### Budget

Past years’ budget information for HIV/AIDS-related research activities throughout the NIH was obtained from NIH budgetary and reporting archives (details provided in the Appendix). Note that a change in programmatic funding reporting led to a substantial reduction in funds reported for HIV therapeutics development from 2017 onward [12,13]. Annual discerned funding values were adjusted to 2017 US dollars based on the consumer price index [14] and are provided in the Appendix (Table A1).

### Approved drugs

Antiretroviral drugs approved by the FDA through 2018 for the treatment of HIV infection were included in this analysis [3].

### Statistical analysis

A series of multiple linear regression models was constructed to test the relationship between funding and ChemDB-based HIV therapeutics research metrics; hypothesis tests on the estimated regression coefficients determined whether the slope of each predictor variable was significantly different from zero. For each regression model, the estimated slope values represent the individual effect of the predictor variable on the response variable of interest when holding the other predictor variables constant. Slope estimates, p-values, and 95% confidence intervals for results reported in the text are provided in the Appendix (Table A5).

## Results

### Therapeutics funding

Much of HIV-related research in the US is funded through the NIH [15]. NIH monies fund a range of activities, including programs for HIV treatment, advocacy, prevention, and research. In 1983, the year HIV was identified, just over $53 million (in 2017 dollars) was budgeted by the NIH for HIV/AIDS research. At its peak in 2004, this budget was over $3.5 billion (Tables 1 and A1). Therapeutics research consumed a large proportion of the NIH’s total HIV/AIDS funding resources early in the epidemic, peaking at 47% of the total budget in 1987 and at a value of over $960M in 2003 (Tables 1 and A1). Therapeutics research funding has remained robust at between one-fifth and one-quarter of the NIH HIV/AIDS budget for the past 13 years (2004–2017, after which programmatic reporting was changed; Table A1), indicating that therapeutics research remains a significant priority.

### Therapeutics activities

To elucidate the influences of funding on research trends, we examined annual HIV therapeutics research activity levels in the context of the NIH’s therapeutics research budget. The research activities analyzed were: (1) the total number of putative HIV-inhibitory compounds described; (2) the number of unique viral or host cellular components investigated as targets for HIV inhibition; (3) the number of publications (or patents) describing a compound(s) with putative activity against HIV; and (4) the total number of target references for HIV inhibition within the publications (or patents). Comprehensive research activity data were gathered from ChemDB and here serve as proxies for activities throughout the therapeutics development field.

**Figure 2.**
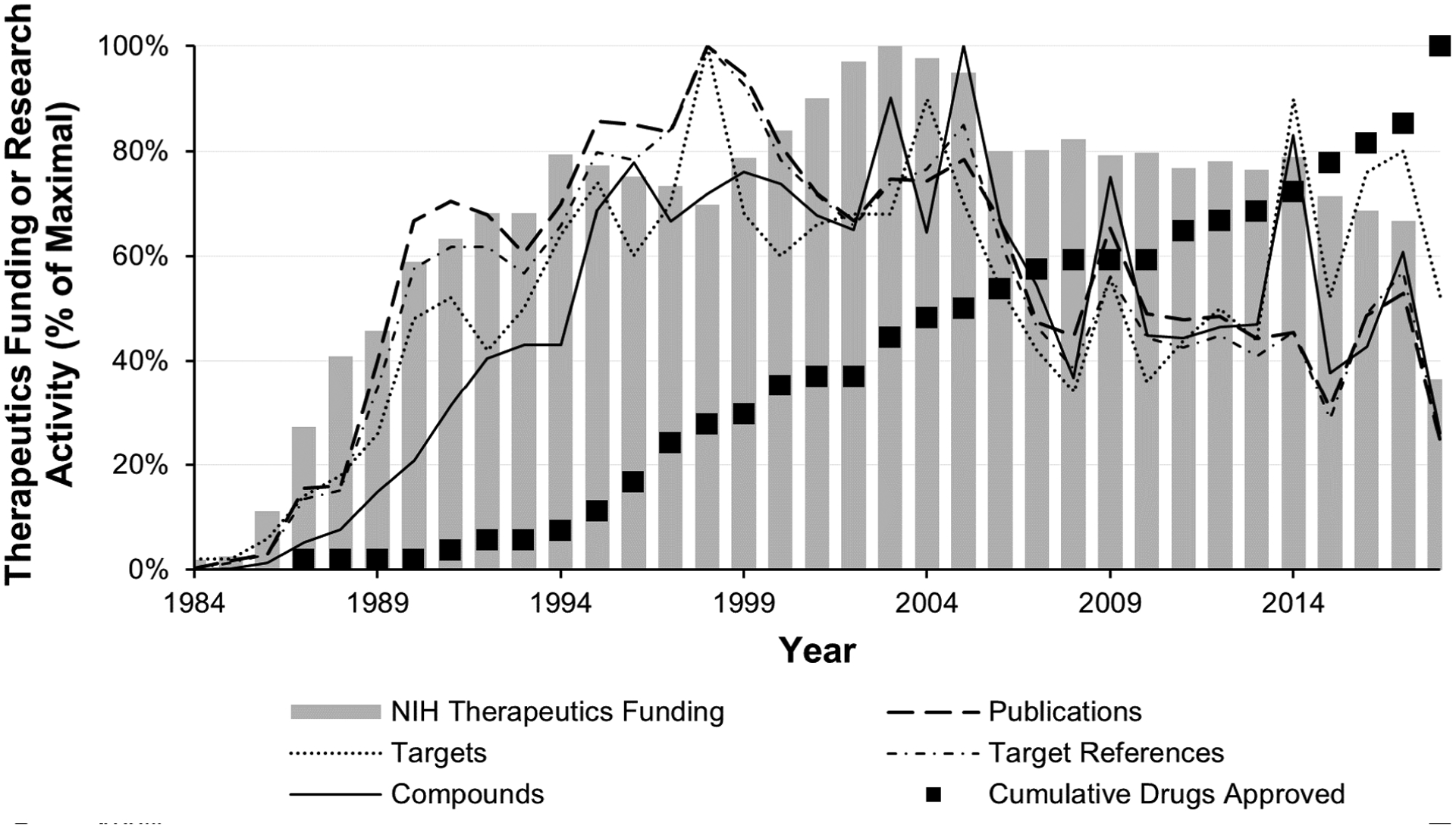
Comparison of Chem-DB-based metrics for research activities with yearly NIH HIV/AIDS therapeutics funding and FDA drug approvals. **Note:** The gray bars indicate the percentage of maximum NIH therapeutics funding per year in 2017 dollars. The black boxes indicate the percentage of the cumulative maximum of FDA-approved drugs reached per year. The yearly statistics from ChemDB (number of publications, number of targets, number of target references, and number of compounds, overlaid lines) are graphed as a percentage of the maximum value for each metric (see Methods). Underlying data are available in Table A1. Note that a change in programmatic funding reporting led to a substantial reduction in funds reported for HIV therapeutics development from 2017 onward [12,13].

On the whole, the four research activity metrics examined followed similar trends (Figure 2 and Table A1): activities expanded through 1998, leveled off between 1998 and 2005, and have generally declined since then. Thus, as NIH HIV therapeutics funding increased swiftly between 1984 and 1994, so too did research outputs; later declines in research outputs mirrored concurrent declines in funding. In fact, funding levels are significantly correlated with the levels of all four research metrics analyzed (all p<0.01, Table A5). Three of the four metrics peaked in 1998, well before the peak of therapeutics funding in 2003, including the annual number of publications/patents entered into ChemDB (355), individual therapeutic inhibitory targets studied (50), and targets referenced within publications (444). Between 1998 and 2002, these proxy measures of research activity declined to approximately two-thirds of their maxima. They increased again between 2003 and 2005, reflecting the 2003 peak in therapeutics research funding. The remaining measure of research activity, the annual number of potential therapeutic compounds published, peaked in 2005 (5,868), also likely reflecting the 2003 peak in therapeutics research funding.

After 2005, all four research activity metrics decreased, reflecting decreases in funding. In 2017, the last full year for which data were available, the research activity metrics lay at 53–80% of their respective maxima; meanwhile, therapeutics funding was at 67% of its maximum. Also notable is the 2014 spike in compounds and target references, the result of a one-year spike in the average number of compounds described per publication – 30 – when no other year’s publications averaged more than 21 (Figure 2 and Table A1), and possibly the result of a transient increase in publications or patents reporting large therapeutics screening efforts. Interestingly, although target references were also high in 2016–2017, these values were not clearly reflected in an elevation in compounds described in those years. Instead, this reflected an uptick in the average number of targets referenced per publication (1.35 vs. an annual average of 1.18, Table A1).

### Drug approvals

First-in-class HIV drug approvals by the FDA through 2018 are indicated in Figure 1 and Table 2 and are discussed in the Appendix; the timeline of additional approvals is provided in Figures 2 and 4. To date, only seven different targets (four viral and three hosts) are represented among the 55 FDA-approved drugs. Looking at the data together, we see no direct effect of the accumulation of treatment options for HIV-infected individuals on either NIH HIV therapeutics research funding or the four therapeutics research activity proxies (all p>0.2, Table A5). Instead, the accumulation of FDA-approved drugs for HIV treatment has proceeded at a slow but generally steady pace. The approval of eight new drugs in 2018 alone is promising; however, five of these are combination therapies composed of drugs previously approved singly. We watch with interest to see whether this is the beginning of a new trend in drug approvals, and whether it will impact research activity levels moving forward.

### Target-specific trends in research activities

Antiviral drugs may inhibit HIV replication via interactions with viral targets or with cellular host targets. According to the data within ChemDB, most described potential targets for inhibition of HIV replication are host cellular components, at 176 of 204 total targets (86%), with the remainder being viral components. Conversely, of the nearly 100,000 anti-HIV compounds registered in ChemDB between 1984 and the first half of 2018, 89% were investigated for inhibitory activity via interactions with viral targets, and only 11% via interactions with host targets. Of the 204 targets identified for potential therapeutic intervention, only 10 (8 viral and 2 host) have commanded enough sustained research interest such that the sum of all potential inhibitory compounds described for each meets or exceeds 1% of the total compounds entered into ChemDB. These 10 targets are listed in Table 2 - along with CD4, for which an FDA-approved therapeutic exists but for which the sum of all potential inhibitory compounds in ChemDB has reached only 0.4% of the ChemDB total (Table A6).

**Figure 3.**
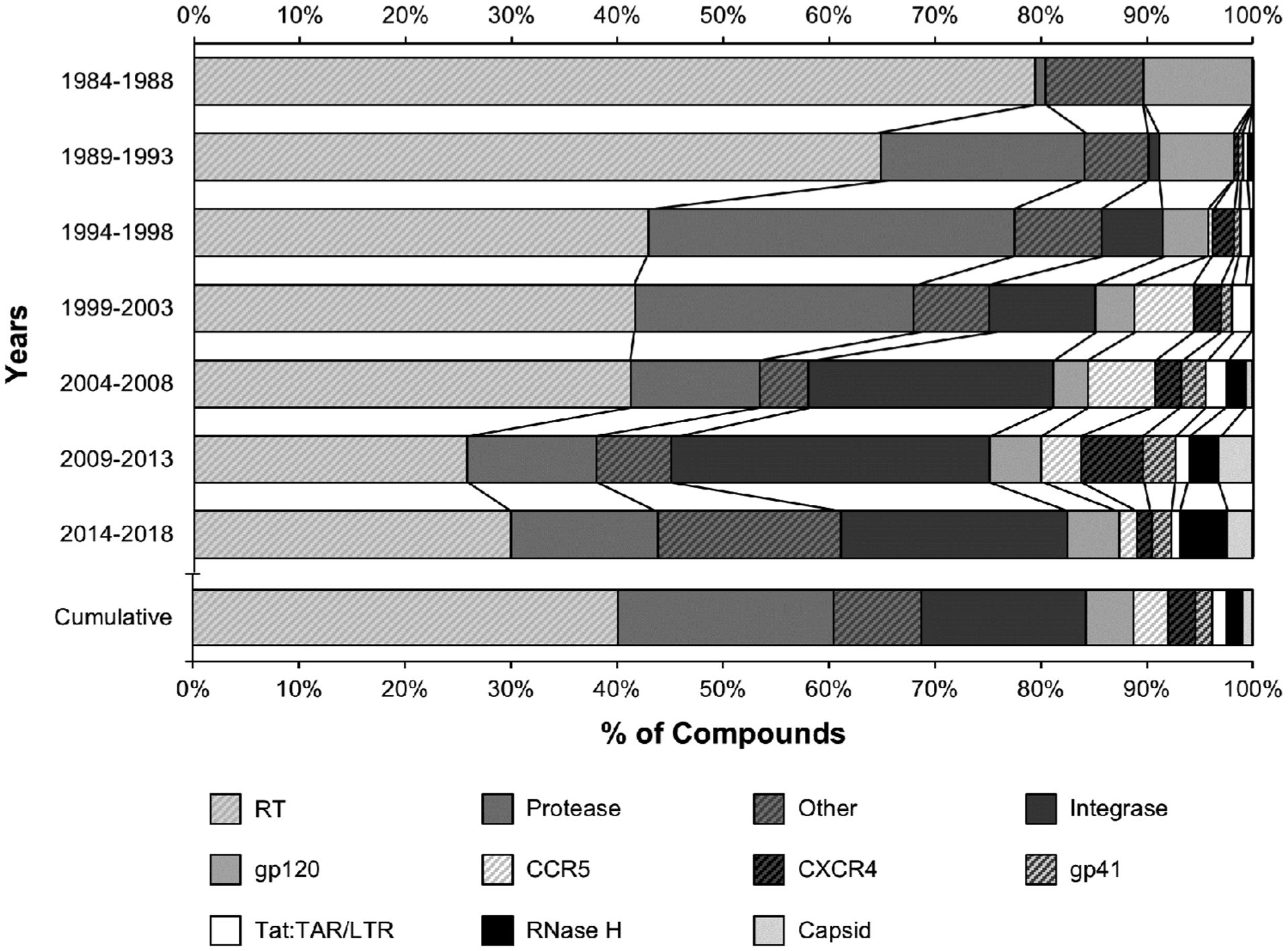
Relative levels of interest in potential therapeutic targets: percentages of target-specific compounds tested in 5-year increments and for all 35 years (1984–2018). **Note:** For each 5-year interval investigated (upper bars) and for the 35-year history of the field (lower bar), the percentages of compounds tested for activity against the top 10 targets for inhibition entered in ChemDB are presented. “Other” indicates the sum of all compounds tested for activity against all targets (viral and host) other than the top 10. Underlying data are available in Tables A2 and A4.

To develop a comprehensible view of target-specific research activities, ChemDB-based proxy metrics were examined in five-year intervals for the 35-year history of the field for the top 10 targets. Figure 3 presents the results of this analysis for the quantities of potential inhibitory compounds described. Given the similarity in results, a parallel analysis of the quantity of references to the targets of these compounds within publications is not shown but provided in the Appendix, along with individual years’ data for both analyses (Figure A1 and Tables A2, A3, and A4).

The overall proportion of potential inhibitory compounds aimed at each target for the entire 35-year history of the field shows that the focus of HIV therapeutic research activities has shifted over time, moving from RT inhibitors in the 1980s to the inclusion of protease inhibitors in the mid- to late-1990s, and then of integrase inhibitors post-2000 (Figure 3). Thus, while RT has dominated therapeutics research activities since the identification of HIV as the etiologic agent behind AIDS, activities aimed at non-RT targets have contributed to over half of all HIV therapeutics research.

Not surprisingly, the proportion of approved drugs for each target mirrors its overall rank in research activity, with 53% (29/55) of approved drugs targeting RT (including 6 RT-only combination therapeutics), 24% (13/55) targeting protease, ~7% targeting integrase (4/55), and the balance targeting fusion, entry, or other processes, and the growing number of multi-class drugs (Figure 3 and Table 2). However, 2018 brought the approval of a therapeutic inhibiting CD4, a target which has historically received a smaller proportion of therapeutics development interest. This points to the exciting possibility of additional future new drug approvals for more of the non-top 10 “other” targets that have received less therapeutics research interest to date.

**Figure 4.**
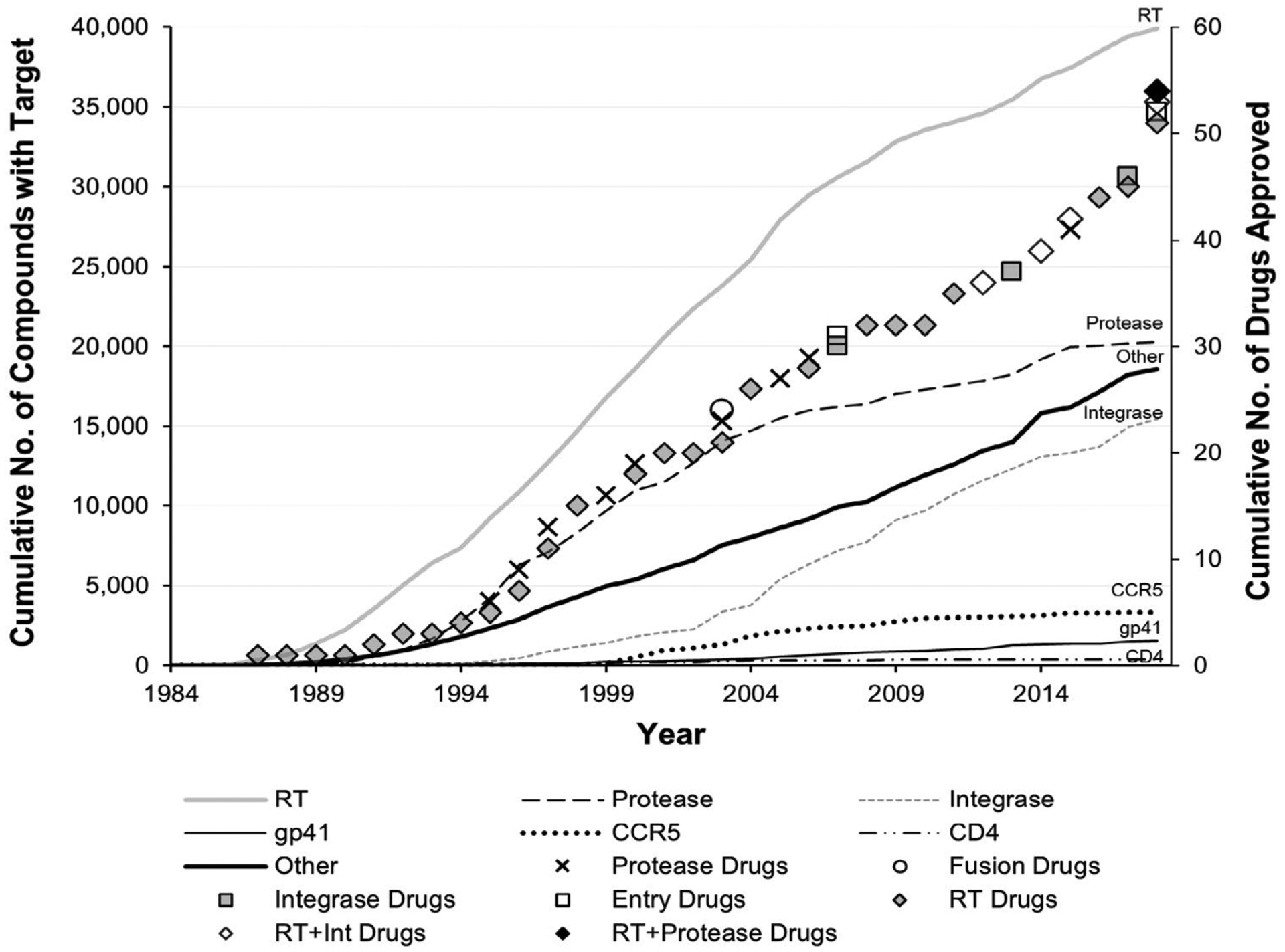
Accumulation of target-specific compounds tested and hiv drug approvals over 35 years (1984–2018). **Note:** The cumulative number of drugs approved by the FDA for all seven targets for which a drug has been approved (symbols) is plotted along with the cumulative number of compounds tested for inhibitory activity against those seven targets, as entered in ChemDB (lines). “Other” indicates the cumulative sum for all targets (viral and host) other than the seven for which HIV drugs have been approved by the FDA. Combination drugs with two or more targets each (RT and integrase, RT and protease, some including cytochrome P450) are depicted by open or black diamonds. Underlying data provided in Tables A2 and A3; research activity data for CD4 provided in Table A6.

Figure 4 provides a different perspective on the 35-year history of HIV therapeutics research from the data gathered from ChemDB. Here, the number of compounds that have been tested for inhibitory activity are viewed cumulatively within a chronology of drug approvals, focusing on the seven targets for which drugs have been approved as of 2018 and with all non-drugged targets now grouped as “other.” This analysis demonstrates that, like their effects on research activities as a whole, the effects of drug approvals for specific targets on the levels of research activities directed at those specific targets are minor (not statistically significant, Table A5). That is, the accumulation of target-specific studies has remained relatively steady in the face of the accumulation of FDA-approved HIV therapeutic options. This may be related to the fact that resistance has been observed quickly following drug introductions, and therefore new drugs are continually needed. Given that Figure 4 also displays a steady increase in the proportion of studies focusing on “other” druggable targets, we conclude that interest in developing truly novel therapeutic options has been and remains high. Even so, the attention to combination therapies in recent years likely reflects the field’s understanding of the individual and epidemiologic advantages of improving patient adherence to treatment regimens. Similar results of a parallel analysis of the cumulative references to the targets of these compounds within publications, again including a drug approval chronology, are presented in the Appendix, along with individual years’ data for both analyses (Figure A2 and Tables A2, A3, and A6).

Inspection of Figures 3 and 4 together reveals that although the number of viral component targets identified has plateaued (due to the small number of HIV proteins available to be therapeutic targets), interest in research activities studying viral targets remain robust. Conversely, although the number of host component targets identified continues to increase, the sum of research activities for those targets remains lower than for viral targets (Tables A2, A3, and A4). This trend may be related to the cytotoxicity hurdles associated with the inhibition of host targets.

## Discussion

In this study, the 35 years of data within the ChemDB HIV, Opportunistic Infection and Tuberculosis Therapeutics Database served as proxies for annual levels of HIV therapeutics “research activities.” Overall, the proxy metrics indicate that interest in developing therapeutics for HIV has remained robust since the advent of the epidemic in the early 1980s, yet varied in response to the availability of research funding (Figure 2).

ChemDB data underscore the difficulties of the therapeutics development process, with the vast majority of investigated compounds failing to progress; while ChemDB houses over 100,000 compounds that were tested for inhibition of HIV, only 55 therapeutic options have progressed to FDA approval (as of 2018). In fact, the percentage of novel compounds published relative to the total number of compounds described, on an annual basis, averaged 82% over the 35-year history of the field (median, Table A2). That is, most compounds failed to progress through multiple years of pre-clinical testing; in response, novel compounds were continually developed for study. Moreover, of those 55 compounds that did become FDA-approved drugs, only 44 are unique - the remainder are combinations of previously approved drugs, further attesting to the difficulties of therapeutics development.

For the top therapeutic targets of RT, protease, and integrase, there appears to be a link between levels of target-specific research and drug approvals (Figures 1, 3 and 4 and Table A2). By the time HIV was identified as a retrovirus, much of the groundwork needed to develop reverse transcriptase inhibitors had been laid [16]. It is no surprise then, that RT was the earliest established drug target for HIV, dominating all research metrics in the 1980s, nor that azidothymidine (AZT) was approved as an HIV therapeutic so quickly (in 1987). The mid- to late-1990s saw the peak of protease research activity, with 46% of all tested compounds targeting protease in 1996 (Table A2). This directly followed the first HIV protease inhibitor drug approval in 1995 (saquinavir) (Figure 1 and Table 2) and preceded four more approvals by 2000 (Figure 4). The rise in interest in integrase in the 2000s may reflect the implementation of HAART in 1996; researchers would likely have been eager to develop drugs targeting enzyme(s) other than RT or protease. Success came in 2007 (raltegravir) not long after the 2005 peak in compounds tested that targeted integrase. Likewise, the approval of a drug targeting CCR5 (maraviroc, 2007) came soon after the peak in CCR5-targeting compounds tested in 2004 (Figure 1 and Table A2). Recently, studies investigating non-top 10 “other” viral and host targets have surged, reaching 17% of published inhibitory compounds in 2014–2018 (Figure 3 and Table A2), instilling hope that new drug approvals are close at hand. However, not all drug approvals show a strong link to target-specific research levels. For example, the peak number of gp41-targeting compounds was seen in 2013, a full decade after the only gp41-targeting drug approval (enfuvirtide, 2003). Moreover, the approval of a drug targeting CD4 in 2018 (ibalizumab) came 15 years after the peak of CD4-targeted compounds tested (2003) (Figure 1 and Table A2).

The approval of even several dozen drugs to date does has not negatively influenced the expanding research interests of the field in terms of identifying new targets for inhibition (p>0.7, Table A5). For example, while research interest in well-established (“top 10”) targets remains strong, an average of six new targets are defined yearly, contributing to the average 26 targets per year studied over the 35-year span of this analysis (Table A7). On the one hand, the pursuit of diversification in the HIV therapeutics research field is promising given HIV’s penchant for developing drug resistance quickly - for five of the seven targets with FDA-approved drugs, including both viral and host targets, drug-resistant viral isolates were observed during clinical trials, prior to final approvals [17–22]. Attaining goals of developing therapeutics that avoid viral resistance and undesirable side effects while promoting regimen adherence may be more successful when a wider net is cast. On the other hand, just seven of the 204 potential targets identified to date are inhibited by FDA-approved drugs. Only time will tell if the net has been cast wide enough already to bear additional therapeutics fruit. Since it has taken an average of over 15 years to successfully develop anti-HIV drugs following target identification (Figure 1 and Table 2), and in one case (CD4), development has taken nearly the full length of the epidemic, we cannot know now whether the next druggable target has already been identified.

## Conclusion and Future Prospects

The fact that therapeutics research funding continues to represent a substantial proportion of the NIH HIV/AIDS budget after 35 years of research, 100,000 potential inhibitory compounds, and 55 FDA-approved drugs indicates that investment in therapeutics research remains a significant priority for patients, healthcare providers, and policymakers. Funding was seen to be the factor with greatest influence on therapeutics research activity levels, although only for RT/AZT did a druggable target move from first identification to FDA-approved drug target quickly. Although the sample size is small and the reasons may be myriad, we noted that the first-in-class drug approvals that occurred after the peak of NIH HIV therapeutics funding (2003) averaged development times that were twice as long (21 years) than those that occurred before/at peak funding (<10 years). Looking toward the future, robust research interest levels are likely to continue to bear fruit at a steady, albeit slow pace, so long as adequate funding is maintained. Over time, these fruits will benefit patients in the form of improved HIV treatment regimens in terms of safety, tolerability, and efficacy. Whether the harvest will happen in a timeframe that supports the UNAIDS 90-90-90 goals and eradication of AIDS by 2030 [23] remains to be seen.

## Limitations

This analysis of HIV therapeutics research activities was limited in scope to those activities for which the data contained within ChemDB could be used as a proxy, and is therefore focused on preclinical data only, with an emphasis on small molecule drug discovery. Analysis of influencing factors considered only NIH funding sources and only drugs approved by the FDA. These choices were made to best leverage a comprehensive set of data to investigate defined, tractable questions. Future analyses in this area might use a broader, international lens to assess the influencing factors of funding and drug approvals, and might be expanded to include clinical research and a focus on drug development rather than drug discovery activities.

## Supplementary Material

Jackson2020_supp

## Figures and Tables

**Table 1.** Historical NIH funding levels for HIV/AIDS research.

Therapeutics Funding Metric	Funding Year					Single-Year Peak	
	1984	1994	2004	2014	2018	Year	Amount
Total HIV/AIDS Funding^‡^	$104M^†^	$2.1B	$3.7B	$3.1B	$2.9B	2004	$3.69B
Therapeutics Funding (TF)	$18M	$768M	$945M	$762M	$352M	2003	$967M
TF as a % of the Total NIH HIV/AIDS Budget	17%	36%	26%	25%	12%	1987	46.8%
No. Drugs Approved (Cumulative 1984-year)	0	4	26	40	55	2018	8

† All funding presented in 2017 dollars. Note that a change in programmatic funding reporting led to a substantial reduction in funds reported for HIV therapeutics development from 2017 onward [12,13].

‡ Total funding includes allocations for the following research categories in addition to Therapeutics: Vaccines, HIV Microbicides, Behavioral and Social Science, Etiology and Pathogenesis, Natural History and Epidemiology, Training, Infrastructure, and Capacity Building, and Information Dissemination.

**Table 2.** Inhibitory targets with FDA-approved HIV drugs through 2018.

Target	Viral/Host	Year Identified as Potential Target	Year of 1^st^ Drug Approval in Class	Number of FDA-Approved Therapeutics (% of All Approved)^†^	
Reverse transcriptase	Viral	1984	1987 NRTI	15 NRTI	6 multi-class combinations (11%)
			1996 NNRTI^‡^	8 NNRTI (42%)	
gp120	Viral	1985		None^§^	
Protease	Viral	1986	1995	13 (24%)	1 combination with RT (2%)
Tat:TAR/LTR	Viral	1986		None	
gp41	Viral	1987	2003	1 (2%)	
Capsid	Viral	1988		None	
Integrase	Viral	1989	2007	4 (7%)	4 combinations with RT (7%)
RNase H	Viral	1989			None
CCR5	Host	1996	2007		1 (2%)
CD4^¶^	Host	1984	2018		1 (2%)
CXCR4	Host	1996			None
Other^⁑^	Host	Various	2014		1 (2%)

† FDA-approved therapeutics for target as of 2018. Total = 55.

‡ NRTI = nucleoside reverse transcriptase inhibitor; NNRTI = Non-nucleoside reverse transcriptase inhibitor.

§ FDA approved first drug in class in 2020, after the period examined by this analysis (see Appendix).

¶ Not a top 10 target.

⁑ Not top 10 targets. A full listing of all other targets is provided in the Appendix (Table A8). FDA-approved drug is a cytochrome P450 inhibitor.

## Abbreviations:

AIDS: Acquired Immunodeficiency Syndrome
AZT: Azidothymidine
FDA: Food and Drug Administration
HAART: Highly Active Antiretroviral Therapy
HIV: Human Immunodeficiency Virus
NIAID: National Institute of Allergy and Infectious Disease
NIH: National Institutes of Health
NRTI: Nucleoside Reverse Transcriptase Inhibitor
NNRTI: Non-nucleoside Reverse Transcriptase Inhibitor
PrEP: Pre-Exposure Prophylaxis
RT: Reverse Transcriptase
SAR: Structure-Activity Relationship
US: United States
WHO: World Health Organization

## References

[1] Barré-Sinoussi Françoise, Chermann Jean-Claude, Rey Fran, and Nugeyre Marie Therese, “Isolation of a T-lymphotropic retrovirus from a patient at risk for acquired immune deficiency syndrome (AIDS).” Science 220, no. 4599 (1983): 868–871. 6189183 10.1126/science.6189183

[2] Gallo Robert C., Salahuddin Syed Z., Popovic Mikulas, and Shearer Gene M., “Frequent detection and isolation of cytopathic retroviruses (HTLV-III) from patients with AIDS and at risk for AIDS.” Science 224, no. 4648 (1984): 500–503. 6200936 10.1126/science.6200936

[3] National Institutes of Health. FDA-Approved HIV Medicines, 2020, accessed.

[4] Larder Brendan A., Darby Graham, and Richman Douglas D.. “HIV with reduced sensitivity to zidovudine (AZT) isolated during prolonged therapy.” Science 243, no. 4899 (1989): 1731–1734. 2467383 10.1126/science.2467383

[5] Rooke Ronald, Tremblay Michel, Soudeyns Hugo, and DeStephano Lucie, “Isolation of drug-resistant variants of HIV-1 from patients on long-term zidovudine therapy.” Aids 3, no. 7 (1989): 411–416. 2504243 10.1097/00002030-198907000-00001

[6] Staszewski Schlomo, Miller Veronica, Rehmet Sibylle, and Stark Thomas, “Virological and immunological analysis of a triple combination pilot study with loviride, lamivudine and zidovudine in HIV-1-infected patients.” AIDS (London, England) 10, no. 5 (1996): F1–7. 10.1097/00002030-199605000-00001 8724034

[7] Guaraldi Giovanni, Cossarizza Andrea, Franceschi Claudio, and Roverato Alberto, “Life expectancy in the immune recovery era: the evolving scenario of the HIV epidemic in northern Italy.” J Acquired Immune Defi Synd 65, no. 2 (2014): 175–181. 10.1097/QAI.0000000000000018 24442223

[8] Harrison Kathleen McDavid, Song Ruiguang, and Zhang Xinjian. “Life expectancy after HIV diagnosis based on national HIV surveillance data from 25 states, United States.” J Acquired Immune Defi Synd 53, no. 1 (2010): 124–130. 10.1097/QAI.0b013e3181b563e7 19730109

[9] Marcus Julia L, Chao Chun R., Leyden Wendy A., and Lanfang Xu, “Narrowing the gap in life expectancy between HIV-infected and HIV-uninfected individuals with access to care.” J Acquired Immune Defi Synd (1999) 73, no. 1 (2016): 39.. 10.1097/QAI.0000000000001014 PMC5427712 27028501

[10] World Health Organization. “HIV drug resistance report 2017.” (Geneva: Organization, World Health, July 2017).

[11] Zorn Kimberley M., Lane Thomas R., Russo Daniel P., and Clark Alex M., “Multiple machine learning comparisons of HIV cell-based and reverse transcriptase data sets.” Mol Pharmaceut 16, no. 4 (2019): 1620–1632. 10.1021/acs.molpharmaceut.8b01297 PMC7702308 30779585

[12] National Institutes of Health Office of AIDS Research. “NIH HIV/AIDS Research Priorities and Guidelines for Determining AIDS Funding, 2015.

[13] National Institutes of Health Office of AIDS Research. “Trans-NIH AIDS Research Budget.” National Institutes of Health.

[14] Oregon State University. “Individual Year Conversion Factor Tables.” Oregon State University, 2019.

[15] U.S. Department of Health and Human Services. “Funding: Budget.” HIV.gov, 2019.

[16] Yarchoan Robert, Mitsuya Hiroaki, Matsushita Shuzo, and Broder Samuel. “Implications of the discovery of HTLV-III for the treatment of AIDS.” Cancer Res 45, no. 9 Supplement (1985): 4685s–4688s. 2410113

[17] Condra Jon H., Schleif William A., Blahy Olga M., and Gabryelski Lori J., “*In vivo* emergence of HIV-1 variants resistant to multiple protease inhibitors.” Nature 374, no. 6522 (1995): 569–571. 7700387 10.1038/374569a0

[18] Emu Brinda, Fessel Jeffrey, Schrader Shannon, and Kumar Princy, “Phase 3 study of ibalizumab for multidrug-resistant HIV-1.” New Eng J Med 379, no. 7 (2018): 645–654. 30110589 10.1056/NEJMoa1711460

[19] Fätkenheuer Gerd, Nelson Mark, Lazzarin Adriano, Konourina Irina, and Hoepelman Andy IM, “Subgroup analyses of maraviroc in previously treated R5 HIV-1 infection.” New Eng J Med 359, no. 14 (2008): 1442–1455. 18832245 10.1056/NEJMoa0803154

[20] Markowitz Martin, Nguyen Bach-Yen, Gotuzzo Eduardo, and Mendo Fernando, “Rapid and durable antiretroviral effect of the HIV-1 integrase inhibitor raltegravir as part of combination therapy in treatment-naive patients with HIV-1 infection: results of a 48-week controlled study.”J Acquired Immune Defi Synd 46, no. 2 (2007): 125–133. 10.1097/QAI.0b013e318157131c 17721395

[21] Wei Xiping, Decker Julie M., Liu Hongmei, and Zhang Zee, “Emergence of resistant human immunodeficiency virus type 1 in patients receiving fusion inhibitor (T-20) monotherapy.” Antimicrobial Agents Chemother 46, no. 6 (2002): 1896–1905. 10.1128/AAC.46.6.1896-1905.2002 PMC127242 12019106

[22] Westby Mike, Lewis Marilyn, Whitcomb Jeannette, and Youle Mike, “Emergence of CXCR4-using human immunodeficiency virus type 1 (HIV-1) variants in a minority of HIV-1-infected patients following treatment with the CCR5 antagonist maraviroc is from a pretreatment CXCR4-using virus reservoir.” J virology 80, no. 10 (2006): 4909–4920. 16641282 10.1128/JVI.80.10.4909-4920.2006 PMC1472081

[23] Joint United Nations Programme on HIV/AIDS, and Joint United Nations Programme on HIV/Aids. “90-90-90: an ambitious treatment target to help end the AIDS epidemic.” Geneva: Unaids (2014).

