# Supplementary material for: A 35-Year Review of Pre-Clinical HIV Therapeutics Research Reported by NIH ChemDB: Influences of Target Discoveries, Drug Approvals and Research Funding
Source: J AIDS Clin Res. Author manuscript; Available in PMC 2020 Dec 23. (PMC7757624)
Supplement: Jackson2020_supp [file NIHMS1653814-supplement-Jackson2020_supp.docx]

**Appendix**

**Contents**

1. **Methods (Literature surveillance methodology, Budget)**
2. **Results**
3. **Figures (A1, A2)**
4. **Data Tables (A1-A8)**
5. **References**

**1. Methods**

**1.1 Literature surveillance methodology**

Online database publication searches using keyword search strings are used to identify sources of data for the ChemDB database on a monthly basis. These searches identify journal articles, meeting abstracts, and patents that describe compounds tested against HIV or *Mycobacterium* species. The data sources are compiled into literature surveillance memos, which are publicly available on the ChemDB website. The data sources are entered into the database if they are *in vivo* or *in vitro* studies that have well-described chemical compounds, natural product extracts, or antibodies, and contain original quantitative biological testing data. These data are then entered into controlled fields in an Oracle database. Although interest is growing in compounds tested for their ability to reactivate latent HIV, these comprise less than 0.4% of the ChemDB data and therefore were not included in this analysis.

**1.2 Budget**

Past years’ budget information for HIV/AIDS-related research activities throughout the NIH was obtained from the archives of the NIH Office of AIDS Research (OAR; reports from 2008-2019) [1-7], the spending history archives of NIH Office of Budget (fiscal year (FY) 1999-2018) [8], the 1992 and 1994 NIH Data Books [9, 10], the NIH Research Portfolio Online Report Tools (RePORT) (FY2011-FY2018) [11], and the 1999 and 2006 HHS Discretionary Funding records [12, 13]. According to RePORT, reported totals for AIDS research include “both extramural and intramural research (including research management and support, Management Fund, and Service & Supply Fund), buildings and facilities, research training, and program evaluation, as well as research on the many HIV-associated co-infections and co-morbidities, including [tuberculosis] TB, hepatitis C, and HIV-associated cancers. It also includes all of the basic science underlying this research” [11]. OAR budget archives provide comprehensive HIV/AIDS categorical budget information for the past 15 years (2004-2018). While funding is broken down into eight categories by the OAR, only therapeutics funding is discussed here. Additional past years’ budgets for therapeutics research were obtained from *The AIDS Research Program of the National Institutes of Health* (1984-1990) [14] and from the National Institutes of Health Fiscal Year 2003 Plan for HIV-Related Research (1997-2002) [15]. Note that a change in programmatic funding reporting led to a substantial reduction in funds reported for HIV therapeutics development from 2017 onward [6, 16]. Annual discerned funding values were adjusted to 2017 US dollars based on the consumer price index [17] and are provided in Table A1.

**2. Results**

**2.1 RT:** By the time HIV was identified as a retrovirus, much of the groundwork needed to develop reverse transcriptase (RT) inhibitors for HIV had already been laid [18]. It is no surprise then, that RT was the earliest established drug target for HIV and that from **1984-1988**, RT was the target of interest in 69% of publications entered into ChemDB (Figure A1 and Tables A3 and A4). Furthermore, nearly 80% of examined potential inhibitory compounds were tested for activity against RT during these five years, including 77% of all potential therapeutic compounds that were considered “novel” for a given year (a compound is “novel” the first time it appears in ChemDB) (Figure 3 and Table A2).

The RT inhibitor AZT (azidothymidine) was approved by the FDA for use as an HIV treatment in 1987, only four years after the identification of HIV as the etiologic agent of AIDS (Figure 1, Table 2). Yet yearly publication counts have remained elevated over the 30 years since, peaking in 1998 at levels nearly five times higher than those seen a decade earlier in 1987-1988, demonstrating sustained interest in the development of RT inhibitors despite the availability of AZT (Table A3). The quick observation of viral resistance to the drug – in 1989 [19, 20] – likely had much to do with this sustained interest. Since those early years, the percentage of described compounds exhibiting anti-HIV activity that were potential RT inhibitors has fallen to as low as 25% in any 5-year interval and as low as 20% in any one year (Figure 3, Table A2). Given that the overall number of publications addressing RT has remained higher than the 1987-1988 level for the past 30 years, the decrease in the proportion of described compounds targeting RT reflects a broadening of the field’s interest. This is supported by the observed increases in the total numbers of both potential inhibitory compounds and the publications describing them for all non-RT targets (Tables A2 and A3). For example, in the **1994-1998** timeframe, twice as many publications reported on potential therapeutics with RT as the target (814) as did with protease (415) (Figure A1 and Tables A3 and A4); however, now less than half of potential therapeutic compounds (43%, 38% of novel compounds) targeted RT (Figure 3 and Tables A2 and A4). These proportions remained steady for the next decade, through 2008 (Figure 3). Relative interest in RT inhibitors has been declining since its peak in 2005, perhaps reflecting the fact that by 2005, the FDA had already approved sixteen reverse transcriptase inhibitors (Figures 4 and A2 and Table A2). In fact, **2009-2013** represents the first time period during which research activities focused on RT did not lead the field in all respects. During this period, only 26% of described compounds targeted RT, while 30% targeted integrase. This shift did not last, however, as during the 2014-2018 time period, RT again led the field with 30% of described compounds targeting it, while only 21% targeted integrase (Figure 3 and Table A2).

**2.2 Protease:** Because of the essential role it plays in HIV replication, protease has been a popular target for HIV drugs since its identification as a potential therapeutic target in 1986 [21] (Figure 1 and Table 2). During the period **1984-1988**, compounds with the potential to inhibit protease constituted 1% of described compounds (Figure 3 and Tables A2 and A4). Then, while RT-directed research activity was decreasing as a proportion of described compounds beginning in **1989-1993**, the broadening of the therapeutics field’s interest was evident in the concomitant increase in potential protease inhibitors, which composed 19% of the compounds (22% of novel compounds) described during this time period (Figure 3 and Tables A2 and A4), a substantive increase from the previous five years.

During **1994-1998**, the average number of compounds described in each publication was substantially higher for protease (15.7, with 13.4 novel compounds) than for RT (10.2, with 7.1 novel compounds) (Tables A2 and A3). In fact, 1996 was the peak of protease research activity, with 46% of all compounds targeting protease (Table A2). This followed on the heels of the first HIV protease inhibitor drug approval in 1995 (saquinavir, Figure 1 and Table 2); four more were approved in quick succession (two in 1997, 1999, and 2000) (Figures 4 and A2). These multiple rapid drug approvals may have contributed to the post-1996 waning of protease-directed research activities. However, again, viral resistance to the drug was observed quickly – here immediately, in 1995 [22] – and this likely had much to do with the maintenance of interest in protease as an inhibitory target beyond this time, albeit at lower levels. During **1999-2003**, over one-quarter of compounds described with potential inhibitory activity against HIV targets were in development as protease inhibitors, while just 12-14% were so described in the following 15 years **(**Figure 3 and Tables A2 and A4).

**2.3 Integrase:** Integrase was shown to be required for HIV replication in 1989 [23, 24] (Figure 1 and Table 2); therapeutics development activities for integrase followed quickly, first recorded in ChemDB in 1991. Compounds targeting integrase surged from 1% of compounds described during **1989-1993** to 6% in **1994-1998** (7% of novel compounds) (Figure 3 and Tables A2 and A4). In 1997, the number of potential integrase inhibitor compounds developed increased to 11% of all compounds (Table A2), although no integrase-targeted therapeutic was approved until 2007 (raltegravir, Figure 1 and Table 2). Unfortunately, viral resistance to the drug was observed immediately in 2007 [25], again likely a contributing factor to the sustained – and even growing – interest in integrase as an inhibitory target beyond this time. From **1999-2003**, one-tenth of compounds described with potential inhibitory activity against HIV targets were being developed as integrase inhibitors. Then, during **2004-2008**, as relative interest in RT and protease inhibitors waned, this number jumped to nearly one-quarter, and to 30% for **2009-2013**. During the most recent period, **2014-2018**, 21% of described compounds targeted integrase (Figure 3 and Tables A2 and A4).

**2.4 Gp120:** The HIV envelope glycoprotein gp120 was identified as a potential drug target in 1985 [26] (Figure 1 and Table 2), and was the focus of initial viral entry inhibitor research. It was the target for 10% of potential inhibitory compounds between **1984-1988** and 7% between **1989-1993**. Compounds with potential inhibitory activity against gp120 did, however, fall to 4% of the total compounds studied during the decade from **1994-2003**. They have since rebounded and have been at 5% of total compounds studied during the **2009-2018** period. Yearly numbers of compounds targeting gp120 have fluctuated widely, with the maximum seen recently in 2017 **(**Figure 3 and Tables A2 and A4). Despite the sustained research interest, no gp120-directed therapeutic was been approved by the FDA through 2018; the first was approved in mid-2020 (fostemsavir), beyond the scope of this analysis.

**2.5 Gp41:** While gp41 was identified as a potential drug target for HIV treatment in 1987 [27], and the first gp41 inhibitor was approved by the FDA in 2003 (enfuvirtide, Figure 1 and Table 2), the extent of research activities focusing on gp41 inhibitors has been relatively less than for other targets. This may be in part due to the extremely rapid development of resistance to this target – mutations were observed during clinical trials of the gp41-targeted viral fusion inhibitor (2002) [28]. Gp41 was represented in ≤2% of described compounds through 2004, and during the **2004-2008,** **2009-2013**, and **2014-2018** time periods was represented in 2-3% of described compounds (Figure 3 and Tables A2 and A4). Notably, interest in gp41 inhibitory compounds peaked at 8% of total compounds in 2013. This is reflected in the number of compounds reported per publication, which surged to 13.9 in 2013, double what it had averaged over the previous nine years (Tables A2 and A3).

**2.6 CCR5:** In 1996, CCR5 was identified as a cofactor in HIV entry [29] (Figure 1 and Table 2); it was the target of <1% of described compounds during **1994-1998 (**Figure 3 and Tables A2 and A4). CCR5 research activities then increased rapidly, with the number of compounds tested with CCR5 as a therapeutic target peaking in 2004 (Tables A2 and A3). For the decade from **1999-2003** and **2004-2008**, CCR5 composed 6% of described compounds in ChemDB (Figure 3 and Tables A2 and A4). During this time, a CCR5-directed therapeutic was approved by the FDA (maraviroc, 2007, Figure 1 and Table 2). As with the other FDA-approved therapeutics, evasion of its therapeutic effects was observed nearly immediately [30-33]. With some variation, CCR5-antagonist research has generally been decreasing since its peak in 2004. During the period **2009-2013**, compounds targeting CCR5 were maintained at 4% of described compounds and fell below 2% of total described compounds in **2014-2018**. Note that CCR5 is one of only two host targets (out of 176) for which research activities have reached levels of at least 1% of total (Figures 3 and A1 and Tables A2, A3, and A4).

**2.7 CXCR4:** Also in 1996, CXCR4 was identified as another cofactor in HIV entry [34] (Figure 1 and Table 2). CXCR4 was the target of 2-3% of described compounds during the fifteen years including the **1994-1998**, **1999-2003**, and **2004-2008** periods. During **2009-2013**, CXCR4 was the target of 6% of described compounds (Figure 3 and Tables A2 and A4). Note that CXCR4 is the other of the two host targets for which research activities have reached levels of at least 1% of total (Figures 3 and A1 and Tables A2, A3, and A4). Unfortunately, despite the sustained research interest, no CXCR4-directed therapeutic has been approved by the FDA to date.

**2.8 RNase H, capsid, and Tat:TAR/LTR:** Although these three viral components were recognized as potential drug targets between 1986 and 1989 (Figure 1 and Table 2) [35-37], they commanded lower levels of interest then the other top ten inhibitory targets. Both **RNase H** and **capsid** were represented in <2% of described compounds until the **2009-2013** and **2014-2018** periods, when compounds targeting either reached 3-4% of described compounds. Compounds targeting **Tat:TAR/LTR** averaged 2% or less of described compounds during all time periods (Figure 3 and Tables A2 and A4). No therapeutics directed at RNase H, capsid, or Tat:TAR/LTR have been approved by the FDA to date.

**2.9 “Other” compounds:** Taken together, additional “other” targets for inhibition represented the focus of 5-9% of all described potential inhibitor compounds from 1984 through 2013; “other” targets represented the focus of 8% of all described potential inhibitor compounds over the entire 35 year study period (Figure 3 and Tables A2 and A4). Recently studies investigating “other” viral and host targets have surged, reaching 17% of published inhibitory compounds in **2014-2018 (**Figure 3 and Tables A2 and A4). In this analysis, “other” targets included CD4, which was identified as a potential target for inhibition very early on in 1984, and for which an inhibitory therapeutic was approved by the FDA many years later in 2018 (ibalizumab, Figure 1 and Table 2). Once again, resistance to the new therapeutic was observed immediately [38]. This approval comes for a lesser-studied target in terms of inhibitory compounds – as noted, the sum of all potential CD4-inhibitory compounds in ChemDB has not reached 1% of the ChemDB total (Table A6) – although CD4 itself has garnered much attention across all of HIV research. One factor keeping the quantity of CD4-targeted inhibitory compounds low in the ChemDB may be that much of the attention on CD4 is focused on antibody-based rather than small molecule therapeutics, which are often not found in ChemDB. This instance serves as a reminder that while the small molecule data within ChemDB and the analyses presented here are proxies for research interest and activity levels, they do not encompass all areas of anti-HIV therapeutics research. Hopefully, the future will bring additional new drug approvals for more of the “other” compounds that have received less research interest to date, as well as molecules that are not recorded in ChemDB. A list of the “other” targets appearing in ChemDB is provided in Table A8.

**3. Figures**


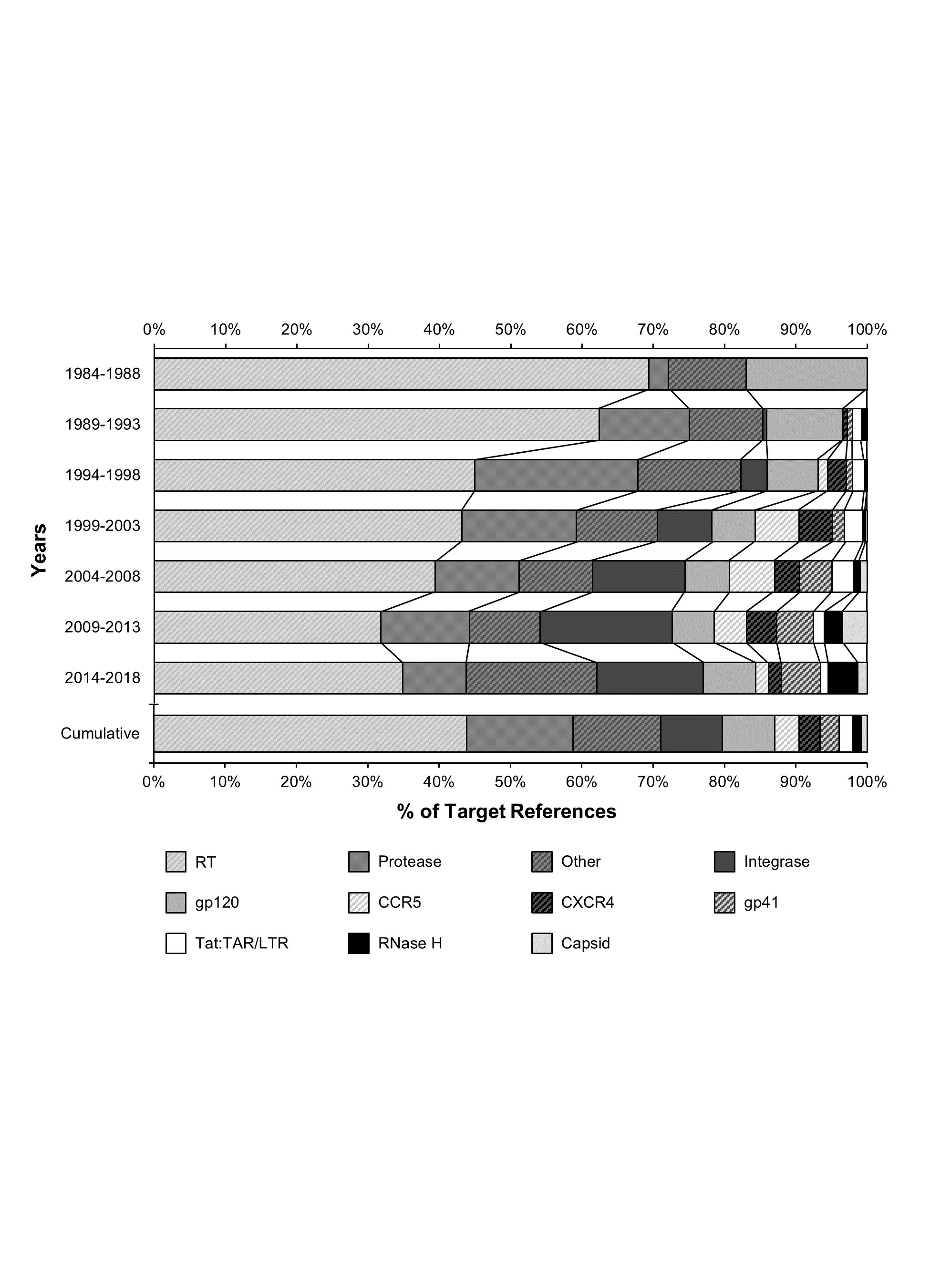


**Figure A1. Relative Levels of Interest in Potential Therapeutic Targets: Percentages of Target-Specific References in Publications Tested in 5-Year Increments and for All 35 Years (1984-2018).** For each 5-year interval investigated (upper bars) and for the 35-year history of the field (lower bar), the percentages of references to the top 10 targets for inhibition entered in ChemDB are presented. “Other” indicates the sum of target references for all targets (viral and host) other than the top 10. Underlying data are available in Table A4.


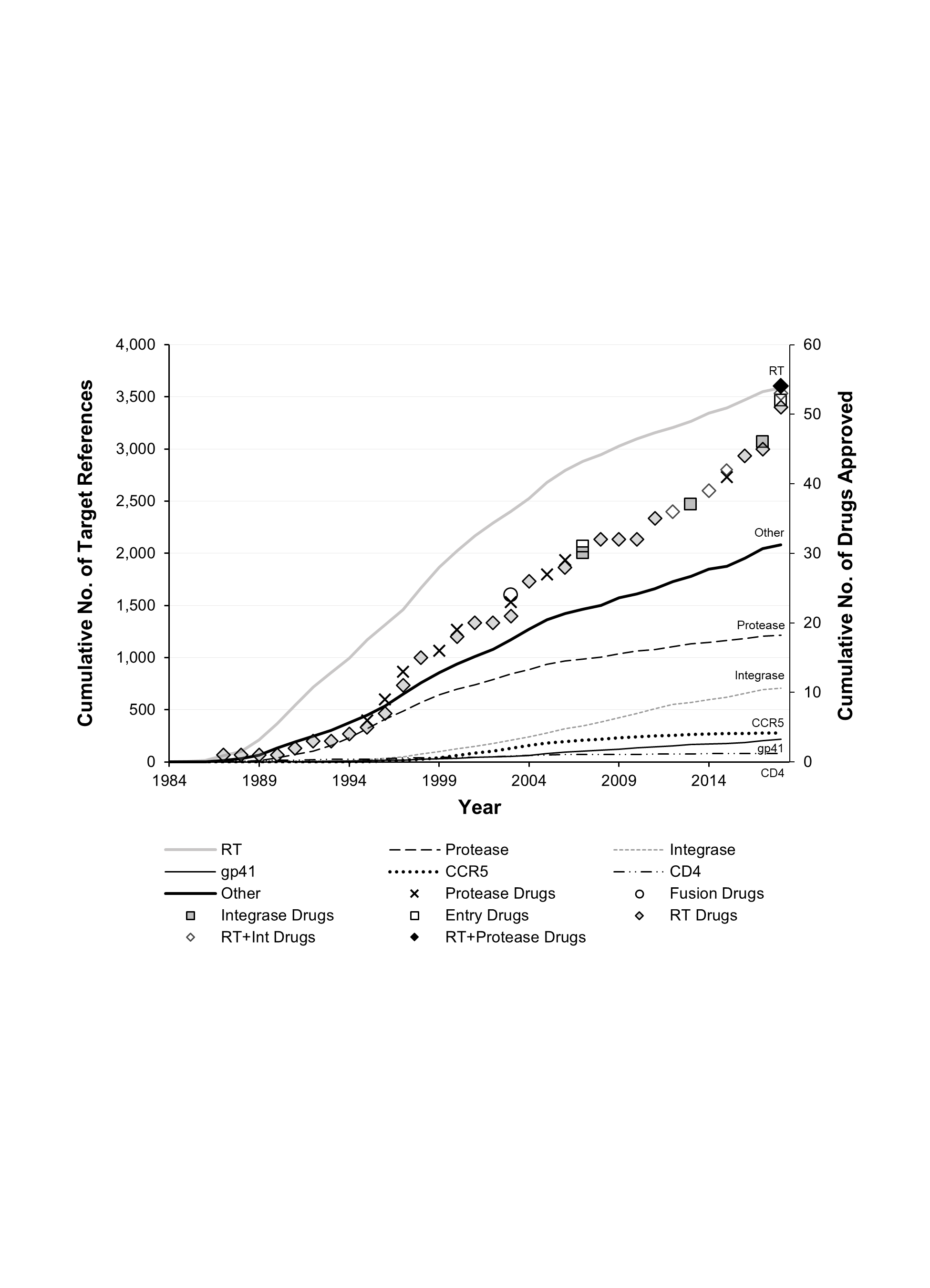


**Figure A2. Accumulation of Target-Specific References in Publications Over 35 Years (1984-2018).** The cumulative number of drugs approved by the FDA for all six targets for which a drug has been approved (symbols) is plotted along with the cumulative number of references to those six targets found in publications, as entered in ChemDB (lines). “Other” indicates the cumulative sum for all targets (viral and host) other than the six for which HIV drugs have been approved by the FDA. Combination drugs with two or more targets each (RT and integrase, RT and protease, some including cytochrome P450) are depicted by open or black diamonds. Underlying data provided in Tables A2 and A3; research activity data for CD4 provided in Table A6.

**4. Data Tables**

**Table A1. Annual NIH HIV/AIDS Therapeutics Funding, Anti-HIV Drugs Approved by FDA, and ChemDB-Based Metrics for Research Activities (1984-2018)**

| Year | Funding Metrics^ | | | No. Drugs Approved by FDA | Research Activity Metrics* | | | |
| --- | --- | --- | --- | --- | --- | --- | --- | --- |
|  | Total NIH OAR HIV/AIDS Budget ($K*) | Therapeutics Budget ($K) | Therapeutics % of Ttotal OAR Budget |  | No. Compounds Entered (Novel) | No. Publications Entered | No. Different Targets Cited | No. References to Targets |
| 1984 | $104,166 | $18,113 | 17% | - | 2 (2) | 1 | 1 | 1 |
| 1985 | $145,187 | $24,674 | 17% | - | 13 (13) | 6 | 1 | 6 |
| 1986 | $301,268 | $107,385 | 36% | - | 79 (75) | 11 | 3 | 13 |
| 1987 | $563,514 | $263,616 | 47% | 1 | 306 (245) | 55 | 7 | 60 |
| 1988 | $891,449 | $394,687 | 44% | 0 | 452 (323) | 57 | 9 | 67 |
| 1989 | $1,239,280 | $441,609 | 36% | 0 | 872 (668) | 142 | 13 | 154 |
| 1990 | $1,389,323 | $569,752 | 41% | 0 | 1,223 (929) | 237 | 24 | 256 |
| 1991 | $1,443,110 | $611,930 | 42% | 1 | 1,840 (1,496) | 250 | 26 | 274 |
| 1992 | $1,650,058 | $658,692 | 40% | 1 | 2,375 (1,806) | 241 | 21 | 274 |
| 1993 | $1,818,147 | $658,481 | 36% | 0 | 2,526 (1,971) | 215 | 25 | 252 |
| 1994 | $2,145,137 | $767,641 | 36% | 1 | 2,527 (1,981) | 248 | 32 | 292 |
| 1995 | $2,144,494 | *$746,887* | *35%* | 2 | 4,033 (3,313) | 304 | 37 | 354 |
| 1996 | $2,204,572 | *$726,100* | *33%* | 3 | 4,566 (3,632) | 302 | 30 | 348 |
| 1997 | $2,291,715 | *$709,686* | *31%* | 4 | 3,908 (3,005) | 297 | 35 | 374 |
| 1998 | $2,410,247 | $674,869 | 28% | 2 | 4,213 (3,177) | 355 | 50 | 444 |
| 1999 | $2,639,824 | *$761,313* | *29%* | 1 | 4,460 (3,128) | 336 | 34 | 412 |
| 2000 | $2,851,738 | $811,381 | 28% | 3 | 4,326 (3,388) | 288 | 30 | 348 |
| 2001 | $3,105,930 | $870,278 | 28% | 1 | 3,978 (2,973) | 255 | 33 | 318 |
| 2002 | $3,406,200 | $938,692 | 28% | 0 | 3,815 (3,106) | 237 | 34 | 292 |
| 2003 | $3,618,102 | $966,711 | 27% | 4 | 5,287 (4,425) | 265 | 34 | 328 |
| 2004 | $3,692,408 | $944,866 | 26% | 2 | 3,787 (2,783) | 264 | 45 | 340 |
| 2005 | $3,659,742 | $918,644 | 25% | 1 | 5,868 (4,959) | 278 | 35 | 377 |
| 2006 | $3,519,037 | $773,034 | 22% | 2 | 3,898 (3,118) | 237 | 27 | 278 |
| 2007 | $3,434,360 | $775,726 | 23% | 2 | 3,197 (2,671) | 168 | 21 | 207 |
| 2008 | $3,335,232 | $795,514 | 24% | 1 | 2,159 (1,899) | 159 | 17 | 171 |
| 2009 | $3,450,617 | $766,355 | 22% | 0 | 4,406 (3,856) | 232 | 28 | 249 |
| 2010 | $3,466,678 | $769,653 | 22% | 0 | 2,629 (2,362) | 174 | 18 | 197 |
| 2011 | $3,332,536 | $741,328 | 22% | 3 | 2,602 (2,418) | 170 | 22 | 189 |
| 2012 | $3,281,264 | $754,130 | 23% | 1 | 2,730 (2,461) | 172 | 25 | 199 |
| 2013 | $3,050,363 | $738,419 | 24% | 1 | 2,760 (2,269) | 157 | 22 | 182 |
| 2014 | $3,082,360 | $761,731 | 25% | 2 | 4,874 (4,682) | 161 | 45 | 202 |
| 2015 | $3,102,438 | $690,011 | 22% | 3 | 2,212 (1,997) | 110 | 26 | 128 |
| 2016 | $3,064,401 | $664,258 | 22% | 2 | 2,509 (2,192) | 173 | 38 | 218 |
| 2017 | $3,000,041 | $645,287 | 22% | 2 | 3,562 (3,254) | 188 | 40 | 253 |
| 2018* | $2,928,030 | $351,901 | 12% | 8 | 1,527 (1,409) | 86 | 26 | 111 |
|  | | | | | | | | |
| Maximum Value Year | 2004 | 2003 | 1987 | 2018 | 2005 | 1998 | 1998 | 1998 |
| *^Funding levels are reported in thousands of 2017 dollars. Values in italics were interpolated from surrounding years’ data.*  **Research metrics for January-June 2018 only.* | | | | | | | | |

**Table A2. Annual Numbers of Compounds Tested for Anti-HIV Activity in ChemDB, 1984-2018**

| Year | Yearly Total No. Compounds (Novel) | No. Yearly Compounds Described for Each Target (Novel) | | | | | | | | | | |
| --- | --- | --- | --- | --- | --- | --- | --- | --- | --- | --- | --- | --- |
|  |  | Viral Targets | | | | | | | | Host Targets | | All Other Targets |
|  |  | Reverse Transcriptase | Protease | Integrase | gp120 | gp41 | Tat:TAR/LTR | Capsid | RNase H | CCR5 | CXCR4 |  |
| 1984 | 2 (2) | 0 | 0 | 0 | 0 | 0 | 0 | 0 | 0 | 0 | 0 | 2 (2) |
| 1985 | 13 (13) | 13 (13) | 0 | 0 | 0 | 0 | 0 | 0 | 0 | 0 | 0 | 0 |
| 1986 | 79 (75) | 66 (62) | 0 | 0 | 6 (6) | 0 | 0 | 0 | 0 | 0 | 0 | 7 (7) |
| 1987 | 306 (245) | 272 (220) | 1 (1) | 0 | 15 (13) | 0 | 0 | 0 | 0 | 0 | 0 | 18 (11) |
| 1988 | 452 (323) | 326 (214) | 7 (6) | 0 | 67 (55) | 0 | 0 | 0 | 0 | 0 | 0 | 52 (48) |
| 1989 | 872 (668) | 711 (534) | 66 (62) | 0 | 55 (37) | 0 | 0 | 0 | 4 (4) | 0 | 0 | 36 (31) |
| 1990 | 1,223 (929) | 886 (638) | 123 (115) | 0 | 80 (52) | 11 (11) | 4 (4) | 0 | 4 (4) | 0 | 0 | 115 (105) |
| 1991 | 1,840 (1,496) | 1,277 (987) | 339 (314) | 1 (1) | 115 (98) | 1 (1) | 15 (15) | 0 | 8 (8) | 0 | 4 (4) | 80 (68) |
| 1992 | 2,375 (1,806) | 1,490 (1,059) | 481 (394) | 53 (53) | 173 (136) | 5 (5) | 8 (6) | 4 (4) | 0 | 0 | 30 (27) | 131 (122) |
| 1993 | 2,526 (1,971) | 1,367 (937) | 693 (606) | 33 (32) | 200 (171) | 20 (19) | 18 (18) | 1 (1) | 15 (14) | 0 | 5 (5) | 174 (168) |
| 1994 | 2,527 (1,981) | 952 (577) | 1,069 (965) | 61 (59) | 149 (114) | 11 (6) | 8 (7) | 0 | 2 (2) | 0 | 66 (62) | 209 (189) |
| 1995 | 4,033 (3,313) | 1,866 (1,356) | 1,444 (1,330) | 148 (147) | 164 (130) | 12 (5) | 10 (8) | 0 | 4 (2) | 4 (4) | 127 (102) | 254 (230) |
| 1996 | 4,566 (3,632) | 1,642 (1,185) | 2,081 (1,748) | 163 (130) | 181 (138) | 52 (46) | 14 (10) | 0 | 28 (28) | 21 (11) | 56 (27) | 328 (309) |
| 1997 | 3,908 (3,005) | 1,850 (1,279) | 816 (644) | 433 (411) | 211 (170) | 19 (16) | 29 (21) | 0 | 1 (1) | 30 (13) | 34 (22) | 485 (428) |
| 1998 | 4,213 (3,177) | 1,960 (1,406) | 1,243 (963) | 307 (244) | 121 (86) | 21 (16) | 124 (120) | 0 | 9 (9) | 26 (7) | 97 (59) | 305 (267) |
| 1999 | 4,460 (3,128) | 2,105 (1,434) | 1,335 (874) | 205 (198) | 222 (186) | 82 (77) | 38 (35) | 0 | 8 (8) | 65 (56) | 117 (55) | 283 (219) |
| 2000 | 4,326 (3,388) | 1,815 (1,256) | 1,272 (1,084) | 413 (350) | 107 (89) | 24 (18) | 55 (51) | 0 | 2 (2) | 376 (342) | 64 (43) | 198 (153) |
| 2001 | 3,978 (2,973) | 1,965 (1,484) | 553 (331) | 272 (251) | 80 (43) | 32 (28) | 9 (9) | 0 | 0 | 457 (262) | 104 (80) | 506 (485) |
| 2002 | 3,815 (3,106) | 1,784 (1,396) | 1,178 (980) | 171 (124) | 195 (184) | 22 (21) | 21 (19) | 0 | 6 (6) | 136 (100) | 115 (100) | 187 (176) |
| 2003 | 5,287 (4,425) | 1,438 (978) | 1,409 (1,217) | 1,116 (1,065) | 206 (157) | 64 (42) | 275 (263) | 14 (14) | 0 | 202 (171) | 154 (125) | 409 (393) |
| 2004 | 3,787 (2,783) | 1,679 (1,168) | 591 (351) | 407 (312) | 153 (120) | 32 (27) | 113 (100) | 6 (6) | 11 (11) | 535 (477) | 23 (17) | 237 (194) |
| 2005 | 5,868 (4,959) | 2,443 (1,885) | 796 (640) | 1,630 (1,556) | 176 (134) | 145 (125) | 105 (94) | 4 (4) | 2 (2) | 296 (258) | 106 (90) | 165 (151) |
| 2006 | 3,898 (3,118) | 1,599 (1,186) | 505 (370) | 950 (854) | 64 (43) | 92 (79) | 58 (57) | 29 (26) | 12 (11) | 173 (122) | 184 (174) | 232 (196) |
| 2007 | 3,197 (2,671) | 1,132 (849) | 206 (139) | 844 (761) | 193 (167) | 81 (64) | 11 (10) | 32 (32) | 271 (271) | 143 (128) | 145 (118) | 139 (132) |
| 2008 | 2,159 (1,899) | 949 (795) | 208 (171) | 557 (520) | 36 (25) | 87 (80) | 92 (90) | 52 (52) | 44 (44) | 55 (50) | 6 (3) | 73 (69) |
| 2009 | 4,406 (3,856) | 1,260 (1,072) | 592 (548) | 1,329 (1,153) | 232 (215) | 44 (37) | 96 (94) | 75 (73) | 27 (26) | 246 (199) | 143 (126) | 362 (313) |
| 2010 | 2,629 (2,362) | 707 (630) | 281 (211) | 624 (536) | 27 (23) | 86 (81) | 0 | 124 (122) | 34 (29) | 176 (168) | 499 (493) | 71 (69) |
| 2011 | 2,602 (2,418) | 512 (430) | 282 (253) | 1,003 (976) | 36 (34) | 64 (55) | 47 (46) | 155 (136) | 181 (177) | 82 (75) | 116 (113) | 124 (123) |
| 2012 | 2,730 (2,461) | 563 (468) | 287 (228) | 889 (820) | 353 (333) | 63 (56) | 22 (22) | 73 (69) | 84 (83) | 36 (32) | 77 (71) | 283 (276) |
| 2013 | 2,760 (2,269) | 862 (735) | 408 (370) | 703 (414) | 88 (81) | 209 (198) | 22 (22) | 61 (51) | 99 (97) | 30 (25) | 53 (53) | 225 (223) |
| 2014 | 4,874 (4,682) | 1,293 (1,175) | 947 (928) | 772 (738) | 54 (54) | 54 (50) | 51 (50) | 277 (277) | 81 (77) | 34 (29) | 45 (45) | 1,266 (1,259) |
| 2015 | 2,212 (1,997) | 690 (548) | 748 (735) | 247 (212) | 39 (36) | 22 (14) | 19 (18) | 24 (24) | 100 (96) | 134 (133) | 55 (50) | 134 (131) |
| 2016 | 2,509 (2,192) | 991 (839) | 80 (54) | 413 (351) | 200 (189) | 40 (33) | 30 (29) | 46 (19) | 158 (151) | 3 (2) | 42 (33) | 506 (492) |
| 2017 | 3,562 (3,254) | 929 (792) | 176 (132) | 1,206 (1,172) | 363 (330) | 108 (86) | 1 (1) | 7 (4) | 240 (226) | 68 (65) | 40 (37) | 424 (409) |
| 2018* | 1,527 (1,409) | 497 (432) | 80 (60) | 506 (499) | 65 (52) | 50 (45) | 14 (14) | 1 (1) | 77 (74) | 2 (0) | 23 (21) | 212 (211) |
|  | | | | | | | | | | | | |
| Total No. Cmpds | 99,521 | 39,891 | 20,297 | 15,456 | 4,426 | 1,553 | 1,309 | 985 | 1,512 | 3,330 | 2,530 | 8,232 |
| % of Total Cmpds | 100% | 40.1% | 20.4% | 15.5% | 4.4% | 1.6% | 1.3% | 1.0% | 1.5% | 3.3% | 2.5% | 8.3% |
| No. Novel Cmpds | 81,986 | 30,019 | 16,824 | 13,947 | 3,701 | 1,341 | 1,233 | 915 | 1,463 | 2,729 | 2,155 | 7,659 |
| % of Novel Cmpds | 100% | 36.6% | 20.5% | 17.0% | 4.5% | 1.6% | 1.5% | 1.1% | 1.8% | 3.3% | 2.6% | 9.3% |
| **Research metrics for January-June 2018 only.* | | | | | | | | | | | | |

**Table A3. Annual Numbers of Publications and Target-Specific References in Publications in ChemDB, 1984-2018**

| Year | Yearly No. Publications | % of 35-Year Total Publications | Yearly No. Target  References | % of 35-Year Total Target References | No. Yearly Publications Citing each Target / No. Yearly References to each Target (in Pub.) | | | | | | | | | | |
| --- | --- | --- | --- | --- | --- | --- | --- | --- | --- | --- | --- | --- | --- | --- | --- |
|  |  |  |  |  | Viral Targets | | | | | | | | Host Targets | | All Other Targets |
|  |  |  |  |  | Reverse Transcriptase | Protease | Integrase | gp120 | gp41 | Tat:TAR/LTR | Capsid | RNase H | CCR5 | CXCR4 |  |
| 1984 | 1 | 0% | 1 | 0% | 0 | 0 | 0 | 0 | 0 | 0 | 0 | 0 | 0 | 0 | 1 |
| 1985 | 6 | 0% | 6 | 0% | 6 | 0 | 0 | 0 | 0 | 0 | 0 | 0 | 0 | 0 | 0 |
| 1986 | 11 | 0% | 13 | 0% | 9 | 0 | 0 | 3 | 0 | 0 | 0 | 0 | 0 | 0 | 1 |
| 1987 | 55 | 1% | 60 | 1% | 44 | 1 | 0 | 8 | 0 | 0 | 0 | 0 | 0 | 0 | 6 |
| 1988 | 57 | 1% | 67 | 1% | 43 | 3 | 0 | 14 | 0 | 0 | 0 | 0 | 0 | 0 | 6 |
| 1989 | 142 | 2% | 154 | 2% | 108 | 9 | 0 | 20 | 0 | 0 | 0 | 1 | 0 | 0 | 15 |
| 1990 | 237 | 3% | 256 | 3% | 155 | 26 | 0 | 36 | 3 | 2 | 0 | 2 | 0 | 0 | 29 |
| 1991 | 250 | 4% | 274 | 3% | 181 | 31 | 1 | 24 | 1 | 3 | 0 | 2 | 0 | 1 | 26 |
| 1992 | 241 | 4% | 274 | 3% | 174 | 34 | 3 | 31 | 1 | 4 | 1 | 0 | 0 | 5 | 20 |
| 1993 | 215 | 3% | 252 | 3% | 137 | 53 | 2 | 19 | 4 | 6 | 1 | 3 | 0 | 1 | 21 |
| 1994 | 248 | 4% | 292 | 4% | 135 | 76 | 4 | 22 | 1 | 4 | 0 | 1 | 0 | 5 | 40 |
| 1995 | 304 | 4% | 354 | 4% | 179 | 85 | 11 | 13 | 2 | 4 | 0 | 2 | 2 | 5 | 44 |
| 1996 | 302 | 4% | 348 | 4% | 144 | 94 | 11 | 34 | 3 | 4 | 0 | 1 | 4 | 9 | 41 |
| 1997 | 297 | 4% | 374 | 5% | 147 | 76 | 15 | 35 | 3 | 7 | 0 | 1 | 10 | 14 | 55 |
| 1998 | 355 | 5% | 444 | 5% | 209 | 84 | 26 | 25 | 6 | 13 | 0 | 1 | 9 | 14 | 39 |
| 1999 | 336 | 5% | 412 | 5% | 198 | 71 | 23 | 25 | 6 | 10 | 0 | 3 | 13 | 20 | 36 |
| 2000 | 288 | 4% | 348 | 4% | 153 | 54 | 28 | 19 | 7 | 9 | 0 | 1 | 23 | 17 | 32 |
| 2001 | 255 | 4% | 318 | 4% | 147 | 44 | 20 | 16 | 7 | 4 | 0 | 0 | 26 | 16 | 31 |
| 2002 | 237 | 3% | 292 | 4% | 122 | 48 | 28 | 15 | 5 | 6 | 0 | 2 | 18 | 11 | 31 |
| 2003 | 265 | 4% | 328 | 4% | 112 | 56 | 31 | 28 | 4 | 15 | 4 | 0 | 25 | 15 | 34 |
| 2004 | 264 | 4% | 340 | 4% | 122 | 41 | 32 | 28 | 10 | 15 | 2 | 2 | 27 | 11 | 33 |
| 2005 | 278 | 4% | 377 | 5% | 154 | 48 | 39 | 27 | 18 | 14 | 1 | 2 | 23 | 17 | 30 |
| 2006 | 237 | 3% | 278 | 3% | 116 | 34 | 43 | 11 | 12 | 7 | 5 | 2 | 14 | 7 | 25 |
| 2007 | 168 | 2% | 207 | 3% | 89 | 18 | 28 | 14 | 12 | 2 | 3 | 2 | 14 | 8 | 17 |
| 2008 | 159 | 2% | 171 | 2% | 60 | 21 | 36 | 6 | 10 | 4 | 3 | 4 | 9 | 5 | 13 |
| 2009 | 232 | 3% | 249 | 3% | 83 | 29 | 42 | 15 | 8 | 8 | 5 | 4 | 13 | 8 | 28 |
| 2010 | 174 | 3% | 197 | 2% | 71 | 26 | 39 | 6 | 12 | 0 | 7 | 3 | 12 | 9 | 12 |
| 2011 | 170 | 2% | 189 | 2% | 59 | 16 | 47 | 9 | 8 | 4 | 7 | 11 | 8 | 8 | 11 |
| 2012 | 172 | 3% | 199 | 2% | 51 | 26 | 40 | 17 | 9 | 2 | 7 | 3 | 7 | 13 | 22 |
| 2013 | 157 | 2% | 182 | 2% | 59 | 29 | 20 | 13 | 15 | 2 | 9 | 5 | 6 | 5 | 19 |
| 2014 | 161 | 2% | 202 | 3% | 75 | 14 | 30 | 6 | 5 | 3 | 3 | 9 | 7 | 5 | 31 |
| 2015 | 110 | 2% | 128 | 2% | 51 | 19 | 20 | 5 | 3 | 2 | 1 | 4 | 4 | 4 | 12 |
| 2016 | 173 | 3% | 218 | 3% | 79 | 16 | 35 | 23 | 9 | 3 | 3 | 7 | 1 | 1 | 40 |
| 2017 | 188 | 3% | 253 | 3% | 78 | 22 | 39 | 24 | 22 | 1 | 4 | 12 | 3 | 4 | 39 |
| 2018* | 86 | 1% | 111 | 1% | 35 | 10 | 12 | 9 | 11 | 1 | 1 | 6 | 2 | 2 | 21 |
|  | | | | | | | | | | | | | | | |
| Total No. Publications | 6,831 |  |  |  | 3,585 | 1,214 | 705 | 600 | 217 | 159 | 67 | 96 | 280 | 240 | 861 |
| % of Total Publications | 100% |  |  |  | 52.5% | 17.8% | 10.3% | 8.8% | 3.2% | 2.3% | 1.0% | 1.4% | 4.1% | 3.5% | 12.6% |
| Total No. Target References | 8,168 |  |  |  | 3,585 | 1,214 | 705 | 600 | 217 | 159 | 67 | 96 | 280 | 240 | 861 |
| % of Total Target References | 100% |  |  |  | 43.9% | 14.9% | 8.6% | 7.3% | 2.7% | 1.9% | 0.8% | 1.2% | 3.4% | 2.9% | 10.5% |
| **Research metrics for January-June 2018 only.* | | | | | | | | | | | | | | | |

**Table A4. Numbers of Compounds Tested for Anti-HIV Activity and Numbers of Target-Specific References in Publications in ChemDB, in 5-Year Increments for All 35 Years (1984-2018)**

| Target | Number of Compounds* | | | | | | | | Number of Target References^ | | | | | | | |
| --- | --- | --- | --- | --- | --- | --- | --- | --- | --- | --- | --- | --- | --- | --- | --- | --- |
|  | 1984-1988 | 1989-1993 | 1994-1998 | 1999-2003 | 2004-2008 | 2009-2013 | 2014-2018* | 1984-2018* | 1984-1988 | 1989-1993 | 1994-1998 | 1999-2003 | 2004-2008 | 2009-2013 | 2014-2018* | 1984-2018* |
| Reverse transcriptase | 677 | 5,731 | 8,270 | 9,107 | 7,802 | 3,904 | 4,400 | 39,891 | 102 | 755 | 814 | 732 | 541 | 323 | 318 | 3,585 |
| Protease | 8 | 1,702 | 6,653 | 5,747 | 2,306 | 1,850 | 2,031 | 20,297 | 4 | 153 | 415 | 273 | 162 | 126 | 81 | 1,214 |
| Integrase | - | 87 | 1,113 | 2,195 | 4,368 | 4,545 | 3,144 | 15,452 | - | 6 | 67 | 130 | 178 | 188 | 136 | 705 |
| gp120 | 88 | 623 | 826 | 810 | 622 | 736 | 721 | 4,426 | 25 | 130 | 129 | 103 | 86 | 60 | 67 | 600 |
| gp41 | - | 37 | 115 | 224 | 437 | 466 | 274 | 1,553 | - | 9 | 15 | 29 | 62 | 52 | 50 | 217 |
| CCR5 | - | - | 81 | 1,236 | 1,202 | 570 | 241 | 3,330 | - | - | 25 | 105 | 87 | 46 | 17 | 280 |
| CXCR4 | - | 39 | 380 | 554 | 464 | 888 | 205 | 2,530 | - | 7 | 47 | 79 | 48 | 43 | 16 | 240 |
| Capsid | - | 5 | - | 14 | 123 | 488 | 355 | 985 | - | 2 | - | 4 | 14 | 35 | 12 | 67 |
| RNase H | - | 31 | 44 | 16 | 340 | 425 | 656 | 1.512 | - | 8 | 6 | 6 | 12 | 26 | 38 | 96 |
| Tat:TAR/LTR | - | 45 | 185 | 398 | 379 | 187 | 115 | 1,309 | - | 15 | 32 | 44 | 42 | 16 | 10 | 159 |
| Other | 79 | 536 | 1,580 | 1,565 | 866 | 1,068 | 2,542 | 8,236 | 16 | 125 | 262 | 193 | 142 | 103 | 170 | 1,005 |
|  | | | | | | | | | | | | | | | | |
| Total Number | 852 | 8,836 | 19,247 | 21,866 | 18,909 | 15,127 | 14,684 | 99,521 | 147 | 1,210 | 1,812 | 1,698 | 1,373 | 1,016 | 912 | 8,168 |
| **Number of total compounds entered into ChemDB as of July 2018 with target. Only those targets where the number of potential inhibitory compounds entered into ChemDB reached 1% of the total are listed individually.*  *^Number of total target references entered into ChemDB as of July 2018 mentioning target. Note that a single publication may mention more than one target.* | | | | | | | | | | | | | | | | |

**Table A5. Results of Statistical Analyses of Relationships between NIH HIV/AIDS Therapeutics Funding Levels, Anti-HIV Drugs Approved by FDA, and ChemDB-Based Metrics for Research Activities (1984-2018)**

| Response Variable | Predictor Variable | Slope Estimate | p-value | 2.5% CI | 97.5% CI |
| --- | --- | --- | --- | --- | --- |
| Number of Compounds Tested | (Intercept) | 44.61 | 0.36 | -52.71 | 141.93 |
|  | Therapeutics funding, % of maximal | 1.01 | 3.14E-05 | 0.59 | 1.42 |
|  | Cum. No. of Drugs Approved | 0.01 | 0.32 | -0.01 | 0.04 |
|  | Year | -0.02 | 0.35 | -0.07 | 0.03 |
|  | | | | | |
| Number of Publications | (Intercept) | -18.10 | 0.67 | -105.22 | 69.03 |
|  | Therapeutics funding, % of maximal | 1.10 | 1.74E-06 | 0.73 | 1.47 |
|  | Cum. No. of Drugs Approved | -0.02 | 0.23 | -0.04 | 0.01 |
|  | Year | 0.01 | 0.67 | -0.03 | 0.05 |
|  | | | | | |
| Number of References to Targets | (Intercept) | 15.16 | 0.73 | -73.58 | 103.89 |
|  | Therapeutics funding, % of maximal | 1.18 | 7.54E-07 | 0.80 | 1.56 |
|  | Cum. No. of Drugs Approved | -0.01 | 0.70 | -0.03 | 0.02 |
|  | Year | -0.01 | 0.73 | -0.05 | 0.04 |
|  | | | | | |
| Number of Different Targets | (Intercept) | -8.23 | 0.86 | -103.63 | 87.18 |
|  | Therapeutics funding, % of maximal | 0.87 | 1.65E-04 | 0.46 | 1.27 |
|  | Cum. No. of Drugs Approved | -0.01 | 0.66 | -0.03 | 0.02 |
|  | Year | 0.00 | 0.86 | -0.04 | 0.05 |
|  | | | | | |
| Pre-2005 Cumulative References to Reverse Transcriptase Inhibitors | (Intercept) | 120.24 | 0.02 | 22.44 | 218.02 |
|  | Cumulative Number of Reverse Transcriptase Inhibitor Drugs Approved | 163.62 | <2e-16 | 150.90 | 176.33 |
|  | | | | | |
| Post-2005 Cumulative References to Reverse Transcriptase Inhibitors | (Intercept) | 1157.88 | 2.29E-03 | 551.09 | 1764.67 |
|  | Cumulative Number of Reverse Transcriptase Inhibitor Drugs Approved | 100.12 | 9.34E-05 | 68.00 | 132.23 |
|  | | | | | |
| *CI, confidence interval* | | | | | |

**Table A6. Annual Numbers of CD4 and Other Non-“Top 10” Compounds Tested for Anti-HIV Activity and Target-Specific References in Publications in ChemDB, 1984-2018**

| Year | Yearly Total No. Compounds (Novel) | No. Yearly Compounds Described for Each Non-Top 10 Target^ (Novel) | | | Yearly No. Publications | % of 35-Year Total  Publications | Yearly No. Target  References | % of 35-Year Total Target References | No. Yearly Publications Citing each Non-Top 10 Target^ / No. Yearly References to each Non-Top 10 Target (in Pub.) | | |
| --- | --- | --- | --- | --- | --- | --- | --- | --- | --- | --- | --- |
|  |  | Host Target | All Other Targets (incl. CD4) | All Other Targets (NOT incl. CD4) |  |  |  |  | Host Target | All Other Targets (incl. CD4) | All Other Targets (NOT incl. CD4) |
|  |  | CD4 |  |  |  |  |  |  | CD4 |  |  |
| 1984 | 2 (2) | 2 (2) | 2 (2) | 0 (0) | 1 | 0% | 1 | 0% | 1 | 1 | 0 |
| 1985 | 13 (13) | 0 (0) | 0 (0) | 0 (0) | 6 | 0% | 6 | 0% | 0 | 0 | 0 |
| 1986 | 79 (75) | 7 (7) | 7 (7) | 0 (0) | 11 | 0% | 13 | 0% | 1 | 1 | 0 |
| 1987 | 306 (245) | 13 (6) | 18 (11) | 5 (5) | 55 | 1% | 60 | 1% | 3 | 6 | 3 |
| 1988 | 452 (323) | 2 (0) | 52 (48) | 50 (48) | 57 | 1% | 67 | 1% | 2 | 6 | 5 |
| 1989 | 872 (668) | 5 (4) | 36 (31) | 31 (27) | 142 | 2% | 154 | 2% | 4 | 15 | 11 |
| 1990 | 1,223 (929) | 15 (15) | 115 (105) | 100 (90) | 237 | 3% | 256 | 3% | 4 | 29 | 25 |
| 1991 | 1,840 (1,496) | 23 (22) | 80 (68) | 57 (46) | 250 | 4% | 274 | 3% | 4 | 26 | 22 |
| 1992 | 2,375 (1,806) | 7 (4) | 131 (122) | 124 (118) | 241 | 4% | 274 | 3% | 2 | 20 | 19 |
| 1993 | 2,526 (1,971) | 7 (6) | 174 (168) | 167 (162) | 215 | 3% | 252 | 3% | 3 | 21 | 18 |
| 1994 | 2,527 (1,981) | 14 (14) | 209 (189) | 195 (175) | 248 | 4% | 292 | 4% | 2 | 40 | 38 |
| 1995 | 4,033 (3,313) | 2 (2) | 254 (230) | 252 (228) | 304 | 4% | 354 | 4% | 2 | 44 | 42 |
| 1996 | 4,566 (3,632) | 38 (36) | 328 (309) | 290 (273) | 302 | 4% | 348 | 4% | 4 | 41 | 37 |
| 1997 | 3,908 (3,005) | 14 (12) | 485 (428) | 471 (416) | 297 | 4% | 374 | 5% | 8 | 55 | 48 |
| 1998 | 4,213 (3,177) | 2 (1) | 305 (267) | 303 (266) | 355 | 5% | 444 | 5% | 2 | 39 | 37 |
| 1999 | 4,460 (3,128) | 7 (5) | 283 (219) | 276 (214) | 336 | 5% | 412 | 5% | 3 | 36 | 34 |
| 2000 | 4,326 (3,388) | 30 (28) | 198 (153) | 168 (125) | 288 | 4% | 348 | 4% | 1 | 32 | 31 |
| 2001 | 3,978 (2,973) | 0 (0) | 506 (485) | 506 (485) | 255 | 4% | 318 | 4% | 0 | 31 | 31 |
| 2002 | 3,815 (3,106) | 3 (3) | 187 (176) | 184 (173) | 237 | 3% | 292 | 4% | 2 | 31 | 29 |
| 2003 | 5,287 (4,425) | 79 (74) | 409 (393) | 330 (319) | 265 | 4% | 328 | 4% | 9 | 34 | 25 |
| 2004 | 3,787 (2,783) | 40 (18) | 237 (194) | 197 (176) | 264 | 4% | 340 | 4% | 6 | 33 | 28 |
| 2005 | 5,868 (4,959) | 5 (1) | 165 (151) | 160 (150) | 278 | 4% | 377 | 5% | 4 | 30 | 26 |
| 2006 | 3,898 (3,118) | 34 (6) | 232 (196) | 198 (190) | 237 | 3% | 278 | 3% | 3 | 25 | 22 |
| 2007 | 3,197 (2,671) | 5 (3) | 139 (132) | 134 (129) | 168 | 2% | 207 | 3% | 2 | 17 | 15 |
| 2008 | 2,159 (1,899) | 0 (0) | 73 (69) | 73 (69) | 159 | 2% | 171 | 2% | 0 | 13 | 13 |
| 2009 | 4,406 (3,856) | 4 (4) | 362 (313) | 358 (309) | 232 | 3% | 249 | 3% | 2 | 28 | 27 |
| 2010 | 2,629 (2,362) | 0 (0) | 71 (69) | 71 (69) | 174 | 3% | 197 | 2% | 0 | 12 | 12 |
| 2011 | 2,602 (2,418) | 1 (1) | 124 (123) | 123 (122) | 170 | 2% | 189 | 2% | 1 | 11 | 10 |
| 2012 | 2,730 (2,461) | 0 (0) | 283 (276) | 283 (276) | 172 | 3% | 199 | 2% | 0 | 22 | 22 |
| 2013 | 2,760 (2,269) | 3 (3) | 225 (223) | 222 (220) | 157 | 2% | 182 | 2% | 3 | 19 | 16 |
| 2014 | 4,874 (4,682) | 1 (1) | 1,266 (1,259) | 1,265 (1,258) | 161 | 2% | 202 | 3% | 1 | 31 | 30 |
| 2015 | 2,212 (1,997) | 1 (1) | 134 (131) | 133 (130) | 110 | 2% | 128 | 2% | 1 | 12 | 11 |
| 2016 | 2,509 (2,192) | 5 (4) | 506 (492) | 501 (488) | 173 | 3% | 218 | 3% | 1 | 40 | 39 |
| 2017 | 3,562 (3,254) | 10 (9) | 424 (409) | 414 (400) | 188 | 3% | 253 | 3% | 1 | 39 | 38 |
| 2018* | 1,527 (1,409) | 2 (2) | 212 (211) | 210 (209) | 86 | 1% | 111 | 1% | 1 | 21 | 20 |
|  | | | | | | | | | | | |
| Total No. Compounds | 99,521 | 381 | 8,232 | 7,851 |  |  |  |  |  |  |  |
| % of Total Compounds | 100% | 0.4% | 8.3% | 7.9% |  |  |  |  |  |  |  |
| No. Novel Compounds | 81,986 | 294 | 7,659 | 7,365 |  |  |  |  |  |  |  |
| % of Novel Compounds | 100% | 0.4% | 9.3% | 9.0% |  |  |  |  |  |  |  |
| Total No. Publications |  |  |  |  | 6,831 |  |  |  | 83 | 861 | 784 |
| % of Total Publications |  |  |  |  | 100% |  |  |  | 1.2% | 12.6% | 11.5% |
| Total No. Target References |  |  |  |  | 8,168 |  |  |  | 83 | 861 | 861 |
| % of Total Target References |  |  |  |  | 100% |  |  |  | 1.0% | 10.5% | 9.6% |
| **Research metrics for January-June 2018 only.*  *^Data for top 10 targets is provided in Tables A2 (compounds) and A3 (publications/target references). The top 10 targets are viral reverse transcriptase, protease, integrase, gp120, gp41, Tat:TAR/LTR, capsid, RNaseH, and host CCR5 and CXCR4.* | | | | | | | | | | | |

**Table A7. Annual Numbers of Total and Novel Compounds Tested for Anti-HIV Activity in ChemDB, 1984-2018**

| Year | Yearly No. Targets Studied | Yearly No. New Targets Studied |
| --- | --- | --- |
|  |  |  |
|  |  |  |
| 1984 | 1 | 1 |
| 1985 | 1 | 1 |
| 1986 | 3 | 1 |
| 1987 | 7 | 4 |
| 1988 | 9 | 4 |
| 1989 | 13 | 5 |
| 1990 | 24 | 11 |
| 1991 | 26 | 11 |
| 1992 | 21 | 5 |
| 1993 | 25 | 6 |
| 1994 | 32 | 6 |
| 1995 | 37 | 11 |
| 1996 | 30 | 6 |
| 1997 | 35 | 7 |
| 1998 | 50 | 20 |
| 1999 | 34 | 8 |
| 2000 | 30 | 5 |
| 2001 | 33 | 8 |
| 2002 | 34 | 3 |
| 2003 | 34 | 6 |
| 2004 | 45 | 16 |
| 2005 | 35 | 4 |
| 2006 | 27 | 1 |
| 2007 | 21 | 1 |
| 2008 | 17 | 2 |
| 2009 | 28 | 4 |
| 2010 | 18 | 0 |
| 2011 | 22 | 3 |
| 2012 | 25 | 1 |
| 2013 | 22 | 1 |
| 2014 | 45 | 18 |
| 2015 | 26 | 3 |
| 2016 | 38 | 9 |
| 2017 | 40 | 7 |
| 2018* | 26 | 4 |
| Total No. Targets | 914 | 204 |
| **Research metrics for January-June 2018 only.* | | |

**Table A8. Full List of Targets (“Top 10” and “Other”) Referenced in Publications in ChemDB, 1984-2018**

| Target | Total No. Publications |  | Target | Total No. Publications |
| --- | --- | --- | --- | --- |
| 26S Proteasome | 2 |  | Cyclophilin A | 19 |
| Acetylcholinesterase | 3 |  | Cytidine kinase | 1 |
| Activator protein 1 | 2 |  | Cytochrome P450 | 1 |
| Adenosine deaminase | 16 |  | Cytochrome P450 1A2 | 1 |
| Adenosine diphosphoribose transferase | 1 |  | Cytochrome P450 2C11 | 1 |
| Adenosine phosphotransferase | 1 |  | Cytochrome P450 2C9 | 1 |
| Adenosylmethionine decarboxylase | 1 |  | Cytochrome P450 2D1 | 1 |
| Alpha-glucosidase | 24 |  | Cytochrome P450 2D6 | 1 |
| Alpha-glycosidase iosidase I | 1 |  | Cytochrome P450 2E1 | 1 |
| Alpha-glycosidase iosidase II | 1 |  | Cytochrome P450 3A4 | 9 |
| Alpha-mannosidase | 5 |  | dCMP deaminase | 1 |
| Annexin A2 | 1 |  | DC-SIGN | 8 |
| Annexin A2 heterotetramer | 1 |  | DEAD-Box DDX1 | 1 |
| Apelin receptor | 2 |  | DEAD-Box DDX3 | 5 |
| Apolipoprotein B mRNA editing enzyme, catalytic polypeptide-like 3G | 3 |  | Deoxycytidine kinase | 4 |
| Aryl hydrocarbon receptor | 1 |  | Deoxyhypusine synthase | 2 |
| Apoptosis inhibitor 5 | 1 |  | Deoxyhypusyl hydroxylase | 2 |
| Ataxia-telangiectasia-mutated kinase | 1 |  | Dihydroorotate dehydrogenase | 2 |
| Beta-glucosidase | 4 |  | Dipeptidyl peptidase-4 | 2 |
| Beta-glucuronidase | 3 |  | DNA-dependent protein kinase | 1 |
| BINDING | 0 |  | Enhancer binding protein 1 | 2 |
| Binding, target not specified | 4 |  | Entry, target not specified | 48 |
| Butyrylcholinesterase | 2 |  | Envelope trimer | 9 |
| Calcineurin | 1 |  | Ets-1 | 1 |
| cAMP dependent protein kinase | 2 |  | Farnesyl transferase | 1 |
| Capsid | 67 |  | Formyl peptide receptor-like 1 | 2 |
| Casein kinase II | 1 |  | Frameshift Stimulating Signal | 3 |
| Cathepsin | 0 |  | Furin | 1 |
| Cathepsin D | 47 |  | Fusion, target not specified | 56 |
| Cathepsin G | 3 |  | Gag | 20 |
| CCR2 | 5 |  | Galactosylceramide | 2 |
| CCR3 | 23 |  | Geranylgeranyl transferase I | 1 |
| CCR4 | 4 |  | Gelatinase | 1 |
| CCR5 | 280 |  | Glucose-6-phosphate dehydrogenase | 1 |
| CCR8 | 2 |  | Glucosidase I | 3 |
| CD130 | 1 |  | Glutathione | 2 |
| CD4 | 83 |  | Gonadotropin-releasing hormone receptor | 1 |
| cGMP dependent kinase | 1 |  | gp120 | 600 |
| Chromosome region maintenance 1 | 1 |  | gp160 | 16 |
| c-Jun N-terminal kinase | 1 |  | gp41 | 217 |
| Colony-stimulating factor 1 receptor | 1 |  | Guanylate kinase | 1 |
| CX3CR1 | 1 |  | Hck | 2 |
| CXCR2 | 1 |  | Heparan sulfate | 5 |
| CXCR4 | 240 |  | Histone deacetylase | 2 |
| Cyclin B | 1 |  | Histone deacetylase 1 | 1 |
| Cyclin T1 | 2 |  | Histone deacetylase 2 | 1 |
| Cyclin-dependent kinase | 4 |  | Histone deacetylase 3 | 1 |
| Cyclin-dependent kinase 1 | 3 |  | Histone deacetylase 4 | 1 |
| Cyclin-dependent kinase 2 | 3 |  | Histone deacetylase 5 | 1 |
| Cyclin-dependent kinase 3 | 1 |  | Histone deacetylase 6 | 1 |
| Cyclin-dependent kinase 4 | 1 |  | Histone deacetylase 7 | 1 |
| Cyclin-dependent kinase 7 | 5 |  | Histone deacetylase 8 | 1 |
| Cyclin-dependent kinase 8 | 1 |  | Histone deacetylase 9 | 1 |
| Cyclin-dependent kinase 9 | 7 |  | Histone deacetylase 10 | 1 |
| Cyclophilin | 2 |  | Histone deacetylase 11 | 1 |
| HIV-1 associated topoisomerase II-beta kinase | 1 |  | Protein phosphatase 1 | 2 |
| HUMAN SPERM | 1 |  | Purine nucleoside phosphorylase | 1 |
| Inosine monophosphate dehydrogenase | 7 |  | REV | 5 |
| Integrase | 702 |  | REV-ERBalpha | 1 |
| Integrase model (Tn5 transposase) | 3 |  | Rev:RRE | 43 |
| Integrin alpha4beta7 | 1 |  | Reverse transcriptase | 3582 |
| Interleukin 6 | 1 |  | Reverse transcriptase model (MMLV Reverse transcriptase) | 3 |
| Interleukin-8 | 2 |  | Rho GTPase | 3 |
| Janus kinase | 1 |  | Ribonucleotide reductase | 25 |
| Kappa-opiod receptor | 1 |  | Ribosome | 13 |
| LEDGF/p75-Integrase | 16 |  | RNA | 2 |
| Leucine aminopeptidase | 2 |  | RNA polymerase II | 1 |
| LIM domain kinase 1 | 2 |  | RNase H | 96 |
| L-type Ca2+ channel | 1 |  | RNase L | 3 |
| Lysate | 1 |  | S-adenosylhomocysteine hydrolase | 8 |
| Matrix metalloproteinase | 1 |  | S-adenosylmethionine decarboxylase | 3 |
| Mechanistic target of rapamycin | 2 |  | Serine-arginine-rich splicing factor 1 | 1 |
| Melanoma inhibitor of apoptosis protein | 1 |  | Sigma-2 receptor | 1 |
| Methionine aminopeptidase | 1 |  | SPC3 | 1 |
| MITOCHONDRIAL DNA SYNTHESIS | 1 |  | Stem-loop 3 RNA | 1 |
| Monocyte chemoattractant protein-1 | 1 |  | Sterile alpha motif domain and HD domain-containing protein 1 | 1 |
| Monocyte chemoattractant protein-3 | 1 |  | T cell receptor beta | 1 |
| Monokine | 2 |  | Tat | 30 |
| Mucosal vascular addressin cell adhesion molecule 1 | 1 |  | Tat:TAR/LTR | 159 |
| Myosin light-chain kinase | 1 |  | Thymidine kinase | 8 |
| Na+/K+/2Cl- Cotransporter | 1 |  | Thymidylate kinase | 2 |
| NCp7 | 44 |  | Thymidylate synthase | 2 |
| NCp7:SL3 | 1 |  | Toll-like receptor 7 | 2 |
| NEF | 4 |  | Toll-like receptor 8 | 1 |
| Neuraminidase | 1 |  | Topoisomerase | 2 |
| N-myristoyltransferase | 14 |  | Topoisomerase I | 3 |
| Nuclear factor-kappa B | 43 |  | Tumor necrosis factor alpha | 17 |
| Nucleocapsid | 14 |  | Tyrosine kinase | 3 |
| Nucleolin | 2 |  | Ubiquitin specific peptidase 7 | 1 |
| Nucleotidase | 2 |  | Uridine kinase | 1 |
| Opioid receptors | 1 |  | VIF | 15 |
| Ornithine decarboxylase | 2 |  | VPR | 5 |
| p6 | 2 |  | VPU | 3 |
| p17 | 9 |  | Viral membrane (lipid bilayer) | 1 |
| P300/CBP-associated factor | 1 |  | Vimentin | 1 |
| p38 Mitogen-activated protein kinase | 2 |  | X-linked inhibitor of apoptosis protein (XIAP) | 1 |
| p56lck tyrosine kinase | 1 |  |  |  |
| P-glycoprotein | 3 |  |  |  |
| Phosphodiesterase 4B2 | 1 |  |  |  |
| Phosphodiesterase type IV | 2 |  |  |  |
| Phosphoribosyl pyrophosphate synthetase | 2 |  |  |  |
| Platelet-activating factor | 1 |  |  |  |
| Poly(ADP-ribose) polymerase 1 | 1 |  |  |  |
| Positive transcription elongation factor b | 4 |  |  |  |
| Prolyl endopeptidase | 1 |  |  |  |
| Protease | 1214 |  |  |  |
| Proteasome | 4 |  |  |  |
| Protein disulfide isomerase | 3 |  |  |  |
| Protein kinase A | 5 |  |  |  |
| Protein kinase C | 28 |  |  |  |

**5. References**

[1] National Institutes of Health Office of AIDS Research. "Trans-NIH AIDS Research Budget." National Institutes of Health, <https://www.oar.nih.gov/sites/default/files/2014_OAR_CJ_Trans-NIH_JF.pdf>.

[2] National Institutes of Health Office of AIDS Research. "Trans-NIH AIDS Research Budget." National Institutes of Health, <https://www.oar.nih.gov/sites/default/files/2015_OARTransNIHAIDSResearchBudget_JF.pdf>.

[3] National Institutes of Health Office of AIDS Research. "Trans-NIH AIDS Research Budget." National Institutes of Health, <https://www.oar.nih.gov/sites/default/files/2016_OAR_Volume_I_final_508.pdf>.

[4] National Institutes of Health Office of AIDS Research. "Strategic Plan Archives." National Institutes of Health, Updated Sep 25, 2017, accessed Apr 19, 2019, <https://www.oar.nih.gov/about/strategic-plan/archives>.

[5] National Institutes of Health Office of AIDS Research. "Trans-NIH AIDS Research Budget." National Institutes of Health, <https://www.oar.nih.gov/sites/default/files/2017_OARTransNIHAIDSResearchBudget_508.pdf>.

[6] National Institutes of Health Office of AIDS Research. "Trans-NIH AIDS Research Budget." National Institutes of Health, <https://www.oar.nih.gov/sites/default/files/2018_OARTransNIHAIDSResearchBudget.pdf>.

[7] National Institutes of Health Office of AIDS Research. "Trans-NIH AIDS Research Budget." National Institutes of Health, <https://www.oar.nih.gov/sites/default/files/FY2019_CJ_Final_04192018_508.pdf>.

[8] National Institutes of Health Office of Budget. "AIDS Obligations by Mechanism, FY 1999 - FY 2018." NIH, Updated March 18, 2019, accessed September 27, 2019, <https://officeofbudget.od.nih.gov/spending_hist.html> and <https://officeofbudget.od.nih.gov/pdfs/FY20/AIDS-Obligations-by-Mechanism-FY-1999-FY-2018.pdf>.

[9] National Institutes of Health. *NIH Data Book: Basic Data Relating to the National Institutes of Health.* Bethesda, MD: National Institutes of Health, 1992.

[10] National Institutes of Health. *NIH Data Book: Basic Data Relating to the National Institutes of Health.* Bethesda, MD: National Institutes of Health, 1994.

[11] National Institutes of Health. "NIH: Research Portfolio Online Reporting Tools (RePORT) " Estimates of Funding for Various Research, Condition, and Disease Categories (RCDC), National Institutes of Health, Updated April 19, 2019, <https://report.nih.gov/categorical_spending.aspx>.

[12] Johnson, Judith A. "AIDS Funding for Federal Government Programs: FY1981-FY1999*."* Congressional Research Service, The Library of Congress (Washington, DC). <https://www.everycrsreport.com/reports/96-293.html>.

[13] Johnson, Judith A., and Sharon Coleman. "AIDS Funding for Federal Government Programs: FY1981-FY2006*."* Congressional Research Service, The Library of Congress (Washington, DC). <https://www.everycrsreport.com/files/20040421_RL30731_8e158c4714d00ee695979cefee0dbebb6f4c85c9.pdf>.

[14] Institute of Medicine (US) Committee to Study the AIDS Research Program of the National Institutes of Health. "Supporting the NIH AIDS Research Program." In *The AIDS Research Program of the National Institutes of Health* Washington, DC: National Academies Press, 1991, Internet, <https://www.ncbi.nlm.nih.gov/books/NBK234085/>.

[15] National Institutes of Health Office of AIDS Research. "NIH Fiscal Year 2003 Plan for HIV-Related Research I: Overview " National Institutes of Health, <http://img.thebody.com/nih/pdfs/research_plan.pdf>.

[16] National Institutes of Health Office of AIDS Research. "NIH HIV/AIDS Research Priorities and Guidelines for Determining AIDS Funding." National Institutes of Health, Updated Aug 12, 2015, <https://grants.nih.gov/grants/guide/notice-files/NOT-OD-15-137.html>.

[17] Oregon State University. "Individual Year Conversion Factor Tables." Oregon State University, Updated August 14, 2018, accessed September 27, 2019, <https://liberalarts.oregonstate.edu/spp/polisci/faculty-staff/robert-sahr/inflation-conversion-factors-years-1774-estimated-2024-dollars-recent-years/individual-year-conversion-factor-table-0>.

[18] Yarchoan, Robert, Hiroaki Mitsuya, Shuzo Matsushita, and Samuel Broder. "Implications of the discovery of HTLV-III for the treatment of AIDS." Cancer Res 45 (Sep 1985): 4685s-88s. <https://www.ncbi.nlm.nih.gov/pubmed/2410113>.

[19] Larder, Brendan A., Graham Darby, and Douglas D. Richman. "HIV with reduced sensitivity to zidovudine (AZT) isolated during prolonged therapy." Science 243 (Mar 31 1989): 1731-4. <https://doi.org/10.1126/science.2467383>. <https://www.ncbi.nlm.nih.gov/pubmed/2467383>.

[20] Rooke, Ronald, Michel Tremblay, Hugo Soudeyns, Lucie DeStephano, et al. "Isolation of drug-resistant variants of HIV-1 from patients on long-term zidovudine therapy. Canadian Zidovudine Multi-Centre Study Group." AIDS 3 (Jul 1989): 411-5. <https://doi.org/10.1097/00002030-198907000-00001>. <https://www.ncbi.nlm.nih.gov/pubmed/2504243>.

[21] Kramer, Richard A., Michael D. Schaber, Anna Marie Skalka, K. Ganguly, et al. "HTLV-III gag protein is processed in yeast cells by the virus pol-protease." Science 231 (Mar 28 1986): 1580-4. <https://doi.org/10.1126/science.2420008>. <https://www.ncbi.nlm.nih.gov/pubmed/2420008>.

[22] Condra, Jon H., William A. Schleif, Olga M. Blahy, Lori J. Gabryelski, et al. "In vivo emergence of HIV-1 variants resistant to multiple protease inhibitors." Nature 374 (Apr 6 1995): 569-71. <https://doi.org/10.1038/374569a0>. <https://www.ncbi.nlm.nih.gov/pubmed/7700387>.

[23] Buchow, Hartmut D., Erwin Tschachler, Robert C. Gallo, and Marvin Reitz, Jr. "HIV-I replication requires an intact integrase reading frame." Haematol Blood Transfus 32 (1989): 402-5. <https://www.ncbi.nlm.nih.gov/pubmed/2560448>.

[24] Starnes, Milbrey C., and Yung-chi Cheng. "Human immunodeficiency virus reverse transcriptase-associated RNase H activity." J Biol Chem 264 (Apr 25 1989): 7073-7. <https://www.ncbi.nlm.nih.gov/pubmed/2468665>.

[25] Markowitz, Martin, Bach-Yen Nguyen, Eduardo Gotuzzo, Fernando Mendo, et al. "Rapid and durable antiretroviral effect of the HIV-1 Integrase inhibitor raltegravir as part of combination therapy in treatment-naive patients with HIV-1 infection: results of a 48-week controlled study." J Acquir Immune Defic Syndr 46 (Oct 1 2007): 125-33. <https://doi.org/10.1097/QAI.0b013e318157131c>. <https://www.ncbi.nlm.nih.gov/pubmed/17721395>.

[26] Robey, W. Gerard, Bijan Safai, Stephen Oroszlan, Larry O. Arthur, et al. "Characterization of envelope and core structural gene products of HTLV-III with sera from AIDS patients." Science 228 (May 3 1985): 593-5. <https://doi.org/10.1126/science.2984774>. <https://www.ncbi.nlm.nih.gov/pubmed/2984774>.

[27] Gallaher, William R. "Detection of a fusion peptide sequence in the transmembrane protein of human immunodeficiency virus." Cell 50 (Jul 31 1987): 327-8. <https://doi.org/10.1016/0092-8674(87)90485-5>. <https://www.ncbi.nlm.nih.gov/pubmed/3496970>.

[28] Wei, Xiping, Julie M. Decker, Hongmei Liu, Zee Zhang, et al. "Emergence of resistant human immunodeficiency virus type 1 in patients receiving fusion inhibitor (T-20) monotherapy." Antimicrob Agents Chemother 46 (Jun 2002): 1896-905. <https://doi.org/10.1128/aac.46.6.1896-1905.2002>. <https://www.ncbi.nlm.nih.gov/pubmed/12019106>.

[29] Dragic, Tatjana, Virginia Litwin, Graham P. Allaway, Scott R. Martin, et al. "HIV-1 entry into CD4+ cells is mediated by the chemokine receptor CC-CKR-5." Nature 381 (Jun 20 1996): 667-73. <https://doi.org/10.1038/381667a0>. <https://www.ncbi.nlm.nih.gov/pubmed/8649512>.

[30] Fatkenheuer, Gerd, Mark Nelson, Adriano Lazzarin, Irina Konourina, et al. "Subgroup analyses of maraviroc in previously treated R5 HIV-1 infection." N Engl J Med 359 (Oct 2 2008): 1442-55. <https://doi.org/10.1056/NEJMoa0803154>. <https://www.ncbi.nlm.nih.gov/pubmed/18832245>.

[31] Pfizer Inc. "MARAVIROC Tablets NDA 22-128. Antiviral Drugs Advisory Committee (AVDAC) Briefing Document." Pfizer Inc., <http://citeseerx.ist.psu.edu/viewdoc/download?doi=10.1.1.371.8425&rep=rep1&type=pdf>.

[32] Moore, John P., and Daniel R. Kuritzkes. "A piece de resistance: how HIV-1 escapes small molecule CCR5 inhibitors." Curr Opin HIV AIDS 4 (Mar 2009): 118-24. <https://doi.org/10.1097/COH.0b013e3283223d46>. <https://www.ncbi.nlm.nih.gov/pubmed/19339950>.

[33] Westby, Mike, Marilyn Lewis, Jeannette Whitcomb, Mike Youle, et al. "Emergence of CXCR4-using human immunodeficiency virus type 1 (HIV-1) variants in a minority of HIV-1-infected patients following treatment with the CCR5 antagonist maraviroc is from a pretreatment CXCR4-using virus reservoir." J Virol 80 (May 2006): 4909-20. <https://doi.org/10.1128/JVI.80.10.4909-4920.2006>. <https://www.ncbi.nlm.nih.gov/pubmed/16641282>.

[34] Feng, Yu, Christopher C. Broder, Paul E. Kennedy, and Edward A. Berger. "HIV-1 entry cofactor: functional cDNA cloning of a seven-transmembrane, G protein-coupled receptor." Science 272 (May 10 1996): 872-7. <https://doi.org/10.1126/science.272.5263.872>. <https://www.ncbi.nlm.nih.gov/pubmed/8629022>.

[35] Dayton, Andrew I., Joseph G. Sodroski, Craig A. Rosen, Wei Chun Goh, et al. "The trans-activator gene of the human T cell lymphotropic virus type III is required for replication." Cell 44 (Mar 28 1986): 941-7. <https://doi.org/10.1016/0092-8674(86)90017-6>. <https://www.ncbi.nlm.nih.gov/pubmed/2420471>.

[36] Fisher, Amanda G., Mark B. Feinberg, Steven F. Josephs, Mary E. Harper, et al. "The trans-activator gene of HTLV-III is essential for virus replication." Nature 320 (Mar 27-Apr 2 1986): 367-71. <https://doi.org/10.1038/320367a0>. <https://www.ncbi.nlm.nih.gov/pubmed/3007995>.

[37] Rossmann, Michael G. "Antiviral agents targeted to interact with viral capsid proteins and a possible application to human immunodeficiency virus." Proc Natl Acad Sci U S A 85 (Jul 1988): 4625-7. <https://doi.org/10.1073/pnas.85.13.4625>. <https://www.ncbi.nlm.nih.gov/pubmed/3133655>.

[38] Emu, Brinda, Jeffrey Fessel, Shannon Schrader, Princy Kumar, et al. "Phase 3 Study of Ibalizumab for Multidrug-Resistant HIV-1." N Engl J Med 379 (Aug 16 2018): 645-54. <https://doi.org/10.1056/NEJMoa1711460>. <https://www.ncbi.nlm.nih.gov/pubmed/30110589>.
